# QiMing granules for diabetic retinopathy: a systematic review and meta-analysis of randomized controlled trials

**DOI:** 10.3389/fphar.2024.1429071

**Published:** 2024-08-22

**Authors:** Yazi Zhang, Menglong Shi, Dehui Peng, Weijie Chen, Yucong Ma, Wenting Song, Yuetong Wang, Haiyin Hu, Zhaochen Ji, Fengwen Yang

**Affiliations:** ^1^ Evidence-Based Medicine Center, Tianjin University of Traditional Chinese Medicine, Tianjin, China; ^2^ Haihe Laboratory of Modern Chinese Medicine, Tianjin, China; ^3^ School of Traditional Chinese Medicine, Tianjin University of Traditional Chinese Medicine, Tianjin, China

**Keywords:** qiming granules, QiMing keli, diabetic retinopathy, systematic review, meta-analysis, randomized controlled trials

## Abstract

**Objective:**

This study aimed to assess the efficacy and safety of QiMing granules (QM) in the treatment of patients with diabetic retinopathy (DR).

**Methods:**

We systematically searched multiple databases, including Pubmed, Embase, Web of Science, Cochrane Library, SinoMed, Chinese National Knowledge Infrastructure (CNKI), Wanfang database, and VIP database. Randomized controlled trials (RCTs) of QM in the treatment of DR were collected, and the search time limit was from the establishment of the database to 27 March 2024. Two independent researchers were involved in literature screening, data extraction, and bias risk assessment. The risk of bias in the included studies was assessed using the Risk of Bias Assessment tool for randomized controlled trials of Cochrane Collaboration 2.0 (RoB 2.0). The main outcomes were the overall efficacy, visual acuity, retinal circulation time, macular thickness. The secondary outcomes were the levels of triglyceride (TG), total cholesterol (TC), low-density lipoprotein cholesterol (LDL-C), high-density lipoprotein cholesterol (HDL-C), and glycated hemoglobin (HbA1c). The adverse events was considered the safety outcome. Review Manager 5.4.1 and Stata 15.1 were used for meta-analysis. Data were pooled by random-effects or fixed-effects model to obtain the mean difference (MD), risk ratio (RR), and 95% confidence interval (CI).

**Results:**

A total of 33 RCTs involving 3,042 patients were included in this study. Overall, we demonstrated that QM had a significant clinical effect on DR. QM alone was superior to conventional treatment (CT) in terms of overall efficacy [RR = 1.45, 95% CI: (1.34, 1.58), *p* < 0.00001, moderate certainty], retinal circulation time [MD = −0.56, 95% CI: (−1.01, −0.12), *p* = 0.01] and macular thickness [MD = −11.99, 95% CI: (−23.15, −0.83), *p* = 0.04]. QM plus CT was superior to CT in terms of overall efficacy [RR = 1.29, 95% CI: (1.24, 1.33), *p* < 0.00001], visual acuity [MD = 0.14, 95% CI: (0.11, 0.17), *p* < 0.00001], macular thickness [MD = −14.70, 95% CI: (−21.56, −7.83), *p* < 0.0001], TG [MD = −0.20, 95% CI: (−0.33, −0.08), *p* = 0.001, moderate certainty], TC [MD = −0.57, 95% CI: (−1.06, −0.07), *p* = 0.02], and LDL-C [MD = −0.36, 95% CI: (−0.70, −0.03), *p* = 0.03]. In terms of safety, the incidence of adverse events in the experimental group was less than that in the control group. The results of the GRADE evidence quality evaluation showed that the evidence quality of outcome indicators was mostly low.

**Conclusion:**

QM can effectively improve overall efficacy, visual acuity, macular thickness, retinal circulation time, and reduce the levels of TG, TC, and LDL-C. However, due to the limited number of studies included, a small sample size, and a lack of high-quality literature, the possibility of publication bias cannot be excluded. Moreover, biases are present due to differences in study design, such as the absence of placebo use in the control group and a predominant use of combined intervention designs in the control group, along with deficiencies in allocation concealment and blinding methods. Therefore, more multi-center, large-sample, and rigorously designed studies are needed to substantiate this conclusion.

**Systematic review registration:**

https://www.crd.york.ac.uk/PROSPERO/#recordDetails, identifier CRD42023465165.

## 1 Introduction

Diabetic retinopathy (DR) is one of the most common multisystem microvascular complications of diabetes mellitus. DR can lead to vision loss and is the leading cause of blindness in adults (Hooper et al., 2012; Yau et al., 2012). In particular, the global age-standardized rate of blindness due to diabetic retinopathy increased by 14.9%–18.5% from 1990 to 2020 (GBD, 2019 Blindness and Vision Impairment Collaborators, 2021). The International Diabetes Federation estimated that there are 463 million people with diabetes between the ages of 20 and 79 years worldwide (IDF Diabetes Atlas, 2024). Retinopathy affects approximately one-third of patients with diabetes in the United States, Europe, and Asia (IDF Diabetes Atlas, 2024; Antonetti et al., 2012; Mysona et al., 2015). According to clinical projections, by 2025, about four million people with diabetes will develop retinopathy, which affects the quality of life of patients (Whiting et al., 2011). Blindness and low vision caused by DR have become a major public health concern (Fundus Disease Group Of Ophthalmological Society Of Chinese Medical Association, 2023; Alamri et al., 2021; Flaxel et al., 2020) and have been defined as the second priority in the prevention of blindness by the World Health Organization (Blindness, 2014). DR has become a disease that endangers human health and seriously affects the quality of life of diabetic patients (Yingmei and Ping, 2024).

DR is caused by vascular changes that exacerbate ischemic and inflammatory states, leading to retinal neovascularization and fibrovascular tissue formation at the vitreoretinal interface (Valdezguerrero et al., 2021). Currently, several methods exist for treating DR in Western medicine, including microvascular circulation protective agents, antivascular endothelial growth factor drugs, hormones, retinal laser photocoagulation, and vitrectomy (Yingmei and Ping, 2024). Among these, antivascular endothelial growth factor drugs are the most commonly used treatment for DR. However, they have shortcomings such as short half-life, frequent injections, poor patient compliance, and high cost (Heier et al., 2012; Jiuzhuo and Chuanghui, 2024; Wells et al., 2016). Laser photocoagulation, another crucial DR treatment strategy, has unavoidable adverse effects (Wang and Lo, 2018). It is an invasive procedure, and the scars it produces can damage the retinal structure and vasculature, leading to increased intraocular pressure and a risk of vitreous hemorrhage (Xiaojing and Yani, 2024). Additionally, laser photocoagulation can result in complications such as color vision loss, visual field defect, and intraocular tissue damage (Li and Zhang, 2019). While Western medicine treatment alone can be effective, disease recurrence and the formation of blood stasis can impede vision recovery (Fuchao, 2014). Hormone therapy and surgical treatment may result in adverse reactions, and the effect of single or combined treatment is limited (Yi and Qiong, 2019). Compared with western medicine, the treatment of DR with traditional Chinese medicine (TCM) has been paid more and more attention, and the effect of traditional Chinese medicine treatment is significant and safe. Previous studies have shown that TCM offers certain benefits in the treatment of DR (Li and Chumei, 2018; Liwei, 2019; Wenbin et al., 2021; Xiaoyan et al., 2019).

With TCM being included in ICD-11, the issue of TCM safety will receive more attention and importance internationally (Zhao et al., 2024; Liu et al., 2024; Author Anonymous, 2019). QiMing granules (QM, SFDA approval number Z20090036) is a new proprietary Chinese medicine formulation developed by Professors Liao Pinzheng and Duan Junguo’s team at Chengdu University of Traditional Chinese Medicine (Xiyu et al., 2020), its main ingredients include *Astragalus aaronii, Pueraria montana* var. *lobata, Rehmannia glutinosa, Lycium chinense*, *Cassia obtusifolia L., Leonurus japonicus Houtt.*, *Typha latifolia L.*, *Whitmania pigra Whitma* (Hai Jie, 2013). For detailed information on QM, please refer to Supplementary Material S1. Notably, *P. montana* var. *lobata,* categorized as the “king medicine”, belongs to the Leguminosae family. The dried root of this plant was used in the formulation. The main active ingredients of the dried root include isoflavones such as 3-hydroxypuerarin, 3-methoxy puerarin, and daidzein (Qin and Lingzhen, 2020). Modern studies have demonstrated that it can improve hemorheology and microcirculation, increase insulin receptor sensitivity, dilate blood vessels, and reduce blood pressure (Hai Jie, 2013; Wen, 2016). *Rehmannia glutinosa*, classified as a “minister drug,” belongs to the metaphysics family. The dried root of this plant was used in the formulation. Its key components include catalpol, mulberry glycoside, pyrodigitol phenylethanol glycoside B1, and other phenylethanol glycosides (Qin and Lingzhen, 2020). Modern studies have demonstrated its hypoglycemic effects (Qin and Lingzhen, 2020; Wen, 2016). *Cassia obtusifolia L.*, serving as an adjuvant, was derived from the dry mature seeds of Cassia, a leguminous plant. Its main representative components include naphthopyranone glycosides such as cassia seed glycoside B2, erythrofuscin-6 murine O-β-gentian glycoside, and cassia seed glycoside C (Qin and Lingzhen, 2020), which have demonstrated antioxidant, retinal cell-protecting, retinal cell apoptosis-inhibiting, blood lipid level-reducing, and hehepatoprotective effects (Pengyue et al., 2020). *Astragalus aaronii* contains astragaloside and astragalus polysaccharides (Hai Jie, 2013), which according to modern studies, contribute to liver protection, blood sugar level reduction, blood lipid level reduction, anti-hypoxia, and immune function enhancement (Hai Jie, 2013; Wen, 2016). *Typha latifolia L.* contains flavonol, typhanthin, and isorhamnetin-3muro-neohesperidin (Chinese Pharmacopoeia Commission, 2015), known for their roles in regulating glucose metabolism, lipid metabolism, and immune inflammation (Jin et al., 2019). *Lycium chinense* contains lycium barbarum polysaccharide, which has been demonstrated to lower blood sugar levels, lower blood lipid levels, and protect the liver and retina (Yanmei et al., 2022). *Leonurus japonicus Houtt.* has a remarkable effect on DR (Xuezhi and Haijiang, 2016). *Leonurus japonicus Houtt.* contains cyclic peptides, triterpenoids, flavonoids and other chemical components. Modern studies have shown that it has anti-hypertensive, antioxidant and anti-inflammatory effects (Penghua et al., 2022). *Whitmania pigra Whitman* contains hirudin,an antithrombotic hormone, and various amino acids and other chemical components. Modern studies have demonstrated its anticoagulant, anti-thrombotic, and anti-inflammation, and edema-reducing effects. QM can delay disease progression and effectively treat DR (Wen, 2016).

QM plays a crucial role in the treatment of DR, and its clinical acceptance continues to grow. Several studies have demonstrated that QM offers a combination of low cost, high efficacy, efficacy safety profile, and favorable cost-utility (Hongchao and Yangyang, 2014; Changsheng et al., 2013). However, previous evaluations have often lacked comprehensive investigations into outcomes. This article comprehensively evaluates the safety and efficacy of QM in treating DR. It is essential to provide a reliable basis for the clinical application among patients with DR, enriching the evidence in the field of TCM research and promoting the clinical application of QM.

## 2 Materials and methods

### 2.1 Study registration

This study was conducted and reported per the Preferred Reporting Project (PRISMA) guidelines for systematic reviews and meta-analysis (Page et al., 2021). The PROSPERO registration number is CRD42023465165.

### 2.2 Search strategy and data organization

Two researchers (ZYZ and PDH) independently searched 8 databases, including CNKI, VIP, Wanfang Database, SinoMed, PubMed, Embase, the Cochrane Library, and Web of Science, for RCTs from inception until 27 March 2024. English search terms such as “Qiming Keli,” “qiming granules,” “Diabetic retinopathy,” “DR,” “Diabetic Retinopathies,” “Retinopathies, Diabetic,” “Retinopathy, Diabetic,” and “Randomized Controlled Trial” were employed. Additionally, Chinese search terms such as “Diabetic Retinopathy” and “qiming granules” were used by combining subject words with free words. Further details on additional search terms and strategies in both Chinese and English, tailored to each specific database,can be found in Supplementary Material S2.

### 2.3 Inclusion criteria

Studies were included if they met the following PICO(S) (participants, intervention, control, outcomes (study designs)) criteria:

1. Participants: Patients were diagnosed with DR by a clinician, following international or national diagnostic criteria (Flaxel et al., 2020). There was no restriction regarding gender, age, or disease duration.
2. Intervention/Comparator: The trial group will used QM as the main intervention or loading treatment regimen, and the control group used conventional treatment (CT), including blood glucose control, Western medicine (calcium dobesilate capsules/dispersive tablets/tablets), and fundus laser treatment. In studies employing a loading design, the trial loading scheme had to be consistent between the two groups within the same study.
3. Outcome:
  a) Primary outcomes: overall efficacy, visual acuity (diopter, D), retinal circulation time (second, S), macular thickness (Micrometre, μm). The criteria for the assessment of overall efficacy can be found in Supplementary Material S3.
  b) Secondary outcomes: triglyceride (TG; millimoles concentration, mmol/L), total cholesterol (TC; millimoles concentration, mmol/L), high-density lipoprotein cholesterol (HDL-C; millimoles concentration, mmol/L), low-density lipoprotein cholesterol (LDL-C; millimoles concentration, mmol/L), glycated hemoglobin (HbA1c; millimoles concentration, mmol/L).
  c) Safety outcome: adverse events.
4. Study design: Studies were publicly available RCTs in either Chinese or English languages.

### 2.4 Exclusion criteria

Studies were excluded if they were conference papers, dissertations, duplicate publications, lacked mention of randomization, were not available in full text, involved other organic eye diseases, or involved clinical diagnosis of proliferative DR.

### 2.5 Literature screening and data extraction

Study screening and data extraction were carried out independently by two investigators (CWJ and MYC) based on the inclusion and exclusion criteria. NoteExpress was used to manage records and eliminated duplicates. During literature screening, titles and abstracts were initially reviewed, and after excluding irrelevant literature, the full text was further reviewed to determine final inclusion. An Excel sheet was created to record data. The extracted data included: (1) sample characteristics: author, publication year, sample size, average age of participants, dose of QM used, and treatment duration; (2) study design: randomization, allocation concealment, and blinding; (3) outcome indicators: overall efficacy, visual acuity, macular thickness, and other results. Disagreements were resolved through discussions with a third investigator (SML). Missing data were retrieved by contacting the authors of each article.

### 2.6 Risk of bias assessment

Two investigators (WYT and SWT) independently evaluated the risk of bias in the included studies and cross-checked their findings. The Cochrane Bias Risk Assessment Tool (Risk of bias tools - RoB 2 tool, 2024; Risk of bias tools, 2024; Flaxel et al., 2020; Sterne et al., 2019) was used to evaluate the quality of the included studies, covering six aspects: (1) randomization process; (2) deviations from the intended interventions; (3) missing outcome data; (4) measurement of outcome; and (5) selection of the reported outcome. Disagreements that arose during the assessments were resolved by discussion with a third investigator (SML). Each item was rated as low risk, high risk, or some concerns.

### 2.7 Statistical analysis

RevMan 5.4.1 and Stata 15.1 software were used for statistical analysis. Continuous variables were expressed as mean difference (MD), while binary variables were expressed as risk ratio (RR), both with 95% confidence intervals (CI). Descriptive analysis was used when only one study was included. The I square (*I* ^*2*^) statistic and *P* vaule (*P*) test were used to assess statistical heterogeneity. When *I* ^*2*^ ≤ 50% or *p* ≥ 0.1, there was not significant heterogeneity, the fixed-effect model was adopted (Higgins et al., 2003). When *I* ^*2*^ > 50% or *p* < 0.1, there was significant heterogeneity, the random-effect model was adopted (Shi et al., 2022). Two-tailed *p* < 0.05 was considered statistically significant. Subgroup analyses were performed based on timing of intervention to investigate possible sources of heterogeneity. Additionally, sensitivity analysis of the pooled results was performed using the one-by-one exclusion method. Funnel plot and Egger’s test were used to determine whether there was publication bias for indicators included in more than 10 studies.

### 2.8 Certainty of evidence

We used the Grading of Recommendation, Assessment, Development, and Evaluation (GRADE) framework to assess the certainty of evidence for each outcome, evaluating five domains: (1) study limitations, assessed based on RoB2.0; (2) consistency, evaluated using *I* ^*2*^ values and the agreement of 95% confidence; (3) indirectness; (4) precision, examined through the optimal data sample size; and (5) publication bias, determined by the number of included studies (Gonzalez-Padilla and Dahm, 2021). Similarly, the certainty of evidence by GRADE was also decided by consensus. Depending on the level of evidence, the certainty was considered to be either high, moderate, low, or very low. The primary outcomes are categorized as critical results, while the secondary outcomes are classified as important results.

## 3 Results

### 3.1 Study screening

**FIGURE 1:**
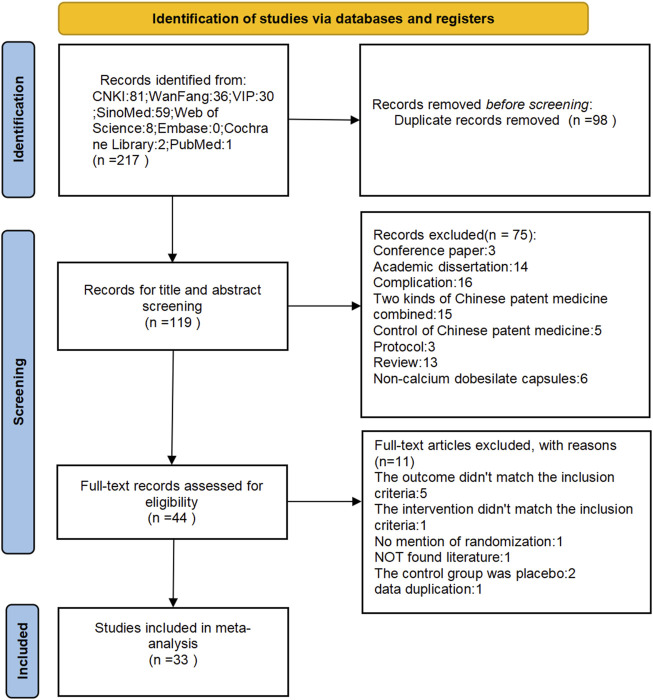
Flow diagram depicting the study screening process.

A total of 217 relevant articles were initially identified through the search process, with 98 duplicates removed, leaving 119 unique articles following rechecking with NoteExpress rechecking. Seventy-five articles were excluded after a preliminary reading of titles and abstracts, and an additional 11 articles were excluded after reading the full text. Finally, 33 relevant articles (Fang et al., 2022; Zheng, 2016; Kong and Dan, 2015; Fan et al., 2018; Yang, 2012; Zang and Yang, 2011; Ge, 2018; Wang et al., 2015; Zhou et al., 2015; Zhang, 2015; Meng et al., 2016; Pang, 2017; Dai et al., 2018; Sui et al., 2014; Zheng et al., 2014; Feng et al., 2016; Yan, 2020; Chen, 2016; Yang et al., 2013; Mu et al., 2018; Zhang, 2013; Zhou, 2017; Wang, 2018; Huang, 2017; Wang et al., 2019; Cao, 2014; Yin, 2018; Zhang et al., 2016; Yue, 2016; Yin and Xiaohua, 2019; Pan et al., 2022; Wu et al., 2015; Wang, 2017) meeting the criteria were included. Figure 1 depicts the screening process for the study.

### 3.2 Study characteristics

Table 1 provides an overview of all study characteristics. For more original information on QM granules, please see Supplementary Material S4. All 33 included studies were RCTs published between 2011 and 2022. The 33 RCTs enrolled a total of 3,042 participants. Six RCTs (Fang et al., 2022; Zheng, 2016; Kong and Dan, 2015; Fan et al., 2018; Yang, 2012; Zang and Yang, 2011) with 546 participants compared QM alone with CT, 27 RCTs (Ge, 2018; Wang et al., 2015; Zhou et al., 2015; Zhang, 2015; Meng et al., 2016; Pang, 2017; Dai et al., 2018; Sui et al., 2014; Zheng et al., 2014; Feng et al., 2016; Yan, 2020; Chen, 2016; Yang et al., 2013; Mu et al., 2018; Zhang, 2013; Zhou, 2017; Wang, 2018; Huang, 2017; Wang et al., 2019; Cao, 2014; Yin, 2018; Zhang et al., 2016; Yue, 2016; Yin and Xiaohua, 2019; Pan et al., 2022; Wu et al., 2015; Wang, 2017) with 2,496 participants compared QM plus CT with CT. The CT included blood glucose control, Western medicine (calcium dobesilate capsules/dispersible tablets/tablets), and fundus laser therapy. The duration of medication ranged from 2 months to 9 months.

**TABLE 1:** Basic characteristics of the included studies.

Study	Age distribution (T/C)	Sample size		Intervention		Course of treatment	Outcome	Adverse event (T/C)
		T (eyes)	C (eyes)	T (dosage)	C			
Zheng et al. (2016)	44.94 ± 5.36/46.72 ± 5.43	47 (74 eyes)	41 (74 eyes)	QM (4.5 g)	CT	6 m	①②③	Not mentioned
Chen (2016)	63.11 ± 5.64/62.05 ± 5.47	45 (90 eyes)	45 (90 eyes)	QM (4.5 g) + CT	CT	3 m	①	Not mentioned
Wang et al. (2015)	52.5 ± 5.3/52.1 ± 5.6	50 (100 eyes)	50 (100 eyes)	QM (4.5 g) + CT	CT	3–6 m	④⑤⑥⑦⑧	The two groups were not adverse reactions
Sui et al. (2014)	50.22 ± 14.82/50.53 ± 11.28	43 (86 eyes)	43 (86 eyes)	QM (4.5 g) + CT	CT	3 m	①④⑤⑥⑦⑧⑨	The two groups were not adverse reactions
Huang (2017)	55.6 ± 4.2/55.9 ± 4.1	63 (126 eyes)	63 (126 eyes)	QM (0.5 g) + CT	CT	3 m	①	Not mentioned
Yin and Xiaohua (2019)	58.69 ± 2.73/58.71 ± 2.68	45 (80 eyes)	45 (78 eyes)	QM (4.5 g) + CT	CT	3 m	①③④	Not mentioned
Zhou (2017)	62.3 ± 7.8/64.1 ± 7.5	42 (84 eyes)	42 (84 eyes)	QM (4.5 g) + CT	CT	3 m	①③④	Not mentioned
Cao (2014)	55.11 ± 2.34/54.37 ± 2.12	50 (100eyes)	50 (100eyes)	QM (4.5 g)+CT	CT	3 m	①③	T: a shadow fluttered before my eyes
								C: a shadow fluttered before my eyes;
								Nausea and diarrhea;
								Significant blood glucose fluctuations
Zhang (2015)	60.03 ± 6.11/60.79 ± 6.42	60 (120eyes)	59 (118eyes)	QM (4.5 g)+CT	CT	3 m	①	Not mentioned
Zhang (2013)	Not mentioned	34 (68 eyes)	34 (68 eyes)	QM (4.5 g) + CT	CT	9 m	①	Not mentioned
Yang et al. (2013)	50.23 ± 7.15/50.94 ± 8.01	35 (70 eyes)	36 (72 eyes)	QM (4.5 g) + CT	CT	6 m	①	Not mentioned
Kong and Dan (2015)	46.77 ± 4.06/45.15 ± 3.26	36 (72 eyes)	36 (72 eyes)	QM (4.5 g)	CT	6 m	①②③④	Not mentioned
Zang and Yang (2011)	Not mentioned	41 (82 eyes)	41 (82 eyes)	QM (4.5 g)	CT	3 m	①	Not mentioned
Yue (2016)	50.67 ± 5.23/49.82 ± 6.17	61 (122 eyes)	59 (118 eyes)	QM (4.5 g) + CT	CT	6 m	①	Not mentioned
Fang et al. (2022)	45.5 ± 1.3/50.0 ± 1.4	51 (51 eyes)	51 (51 eyes)	QM (4.5 g)	CT	6 m	①	T: proliferative diabetic retinopathy; renal injury
								C: proliferative diabetic retinopathy; liver damage; renal injury
Yang (2012)	65/65.5	54 (108 eyes)	54 (108 eyes)	QM (4.5 g)	CT	3 m	①	Not mentioned
Zheng et al. (2014)	55.2 ± 4.7/50.4 ± 3.1	15 (30 eyes)	15 (30 eyes)	QM (4.5 g) + CT	CT	6 m	①	Not mentioned
Meng et al. (2016)	Not mentioned	21 (38 eyes)	21 (36 eyes)	QM (4.5 g) + CT	CT	6 m	①	Not mentioned
Wang (2018)	57.8 ± 6.2/58.4 ± 7.5	44 (88 eyes)	44 (88 eyes)	QM (4.5 g) + CT	CT	3 m	①	Not mentioned
Feng et al. (2016)	55.26 ± 6.29/55.89 ± 6.13	42 (84 eyes)	41 (82 eyes)	QM (4.5 g) + CT	CT	3 m	④⑤⑥⑦⑧⑨	Not mentioned
Wang et al. (2019)	66.7 ± 6.2/66.8 ± 6.3	52 (104 eyes)	48 (96 eyes)	QM (4.5 g) + CT	CT	6 m	①	The two groups were not adverse reactions
Yan (2020)	56.65 ± 4.02/56.96 ± 4.59	41 (41 eyes)	41 (41 eyes)	QM (4.5 g) + CT	CT	2 m	①④⑤⑥⑦⑧⑨	T: proliferative diabetic retinopathy; liver damage
								C: proliferative diabetic retinopathy; liver damage; renal injury
Wang (2017)	54.5 ± 4.8/54.3 ± 4.9	47 (47 eyes)	47 (47 eyes)	QM (4.5 g) + CT	CT	3 m	①	T: epigastric discomfort
								C:None
Ge (2018)	51.25 ± 3.64/50.87 ± 3.71	53 (106 eyes)	53 (106 eyes)	QM (4.5 g) + CT	CT	6 m	①②③	Not mentioned
Yin (2018)	54.63 ± 5.28/55.27 ± 5.42	50 (100 eyes)	46 (92 eyes)	QM (4.5 g) + CT	CT	3 m	①⑤⑥⑦⑧	The two groups were not adverse reactions
Wu et al. (2015)	57.54	106 (212 eyes)	92 (184 eyes)	QM (4.5 g) + CT	CT	3 m	①	Not mentioned
Mu et al. (2018)	51.32 ± 5.24/50.48 ± 5.32	63 (126 eyes)	63 (126 eyes)	QM (4.5 g) + CT	CT	6 m	①	Not mentioned
Pang (2017)	48.2 ± 6.7/47.6 ± 6.5	45 (90 eyes)	45 (90 eyes)	QM (4.5 g) + CT	CT	6 m	①⑤⑥⑦⑧	Not mentioned
Dai et al. (2018)	69.3 ± 1.6/67.5 ± 1.8	31 (62 eyes)	31 (62 eyes)	QM (4.5 g) + CT	CT	3 m	①③	T: a shadow fluttered before my eyes
								C: a shadow fluttered before my eyes; Nausea and diarrhea
Pan et al. (2022)	51.26 ± 8.35/50.98 ± 9.64	47 (47 eyes)	47 (47 eyes)	QM (4.5 g) + CT	CT	6 m	①	Not mentioned
Zhou et al. (2015)	Not mentioned	30 (40 eyes)	30 (36 eyes)	QM (4.5 g) + CT	CT	3 m	①	Not mentioned
Fan et al. (2018)	48.6 ± 5.1/47.5 ± 4.9	47 (60eyes)	47 (60eyes)	QM (4.5 g)	CT	6 m	①②③	T:liver damage; renal injury
								C:proliferative diabetic retinopathy; liver damage; renal injury
Zhang et al. (2016)	Not mentioned	45 (90eyes)	46 (92eyes)	QM (4.5 g) + CT	CT	6 m	①	Not mentioned

Abbreviations: T, treatment group; C, control group; QM: QiMing granules; CT, conventional therapy; m: months; ①, Overall efficacy; ②, Retinal circulation time; ③, Macular thickness; ④, Visual acuity; ⑤, Triglyceride (TG); ⑥, Total cholesterol (TC); ⑦, High-density lipoprotein cholesterol (HDL-C); ⑧, Low-density lipoprotein cholesterol (LDL-C); ⑨, Glycated hemoglobin (HbA1c).

### 3.3 Quality assessment

**FIGURE 2:**
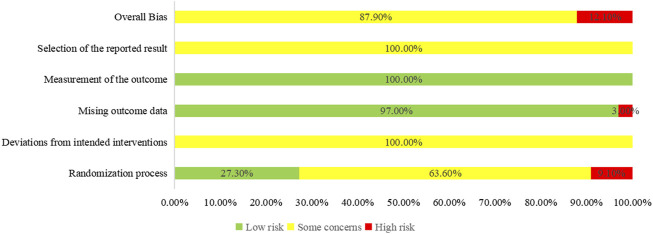
Risk of bias summary.

**FIGURE 3:**
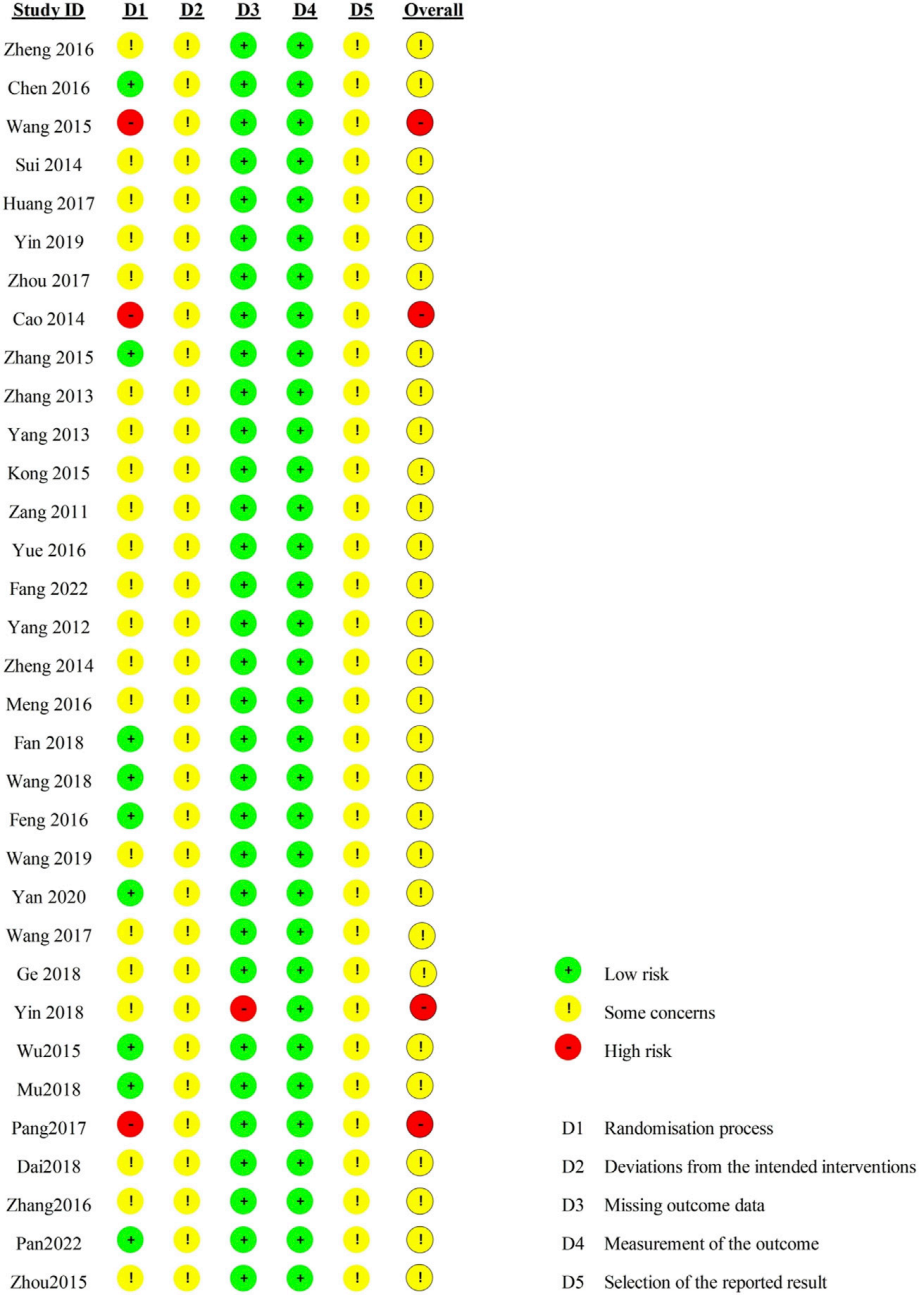
Risk of graph.

Regarding the “randomization process”, there are 29 studies reported comparability of baseline data the two groups, and with 12 studies reported correct randomization methods. But there are 3 studies (Pang, 2017; Wang et al., 2015; Cao, 2014) of them assessed as “high risk” because they reported the wrong method of randomization. The remaining 7 studies (Fan et al., 2018; Pan et al., 2022; Mu et al., 2018; Wang, 2018; Chen, 2016; Zhang, 2015; Wu et al., 2015) using random number table method, 1 study (Feng et al., 2016) using a simple random method, and 1 study (Yan, 2020) using a random lottery method. Therefore, we assessed them as “low risk”. In contrast, the remaining 21 studies were assessed as having “some concerns” due to lack of a specific randomization strategy or no mention of allocation. Furthermore, the trials included no information about participant blinding, outcome assessment, or allocation concealment. We, therefore, rated the “deviations from the intended interventions” as “some concerns”. Included in the study of data has one information is missing (Yin, 2018), we rated it as “high risk”, the rest of the research data are complete and are “low risk”. “Measurement of the outcome” were assessed as “low risk” because the evaluation criteria of outcome measures between the two groups were reasonable in all the studies. Included in the study reported all the expected result. However, the “selective reporting” of all studies was assessed as “some concerns” due to the lack of pre-published study protocols. In general, all studies have some methodological issues. Figures 2, 3 provide an overview of the results of the methodological quality assessment.

### 3.4 Meta-analysis results

#### 3.4.1 Main outcomes

##### 3.4.1.1 Overall efficacy

**FIGURE 4:**
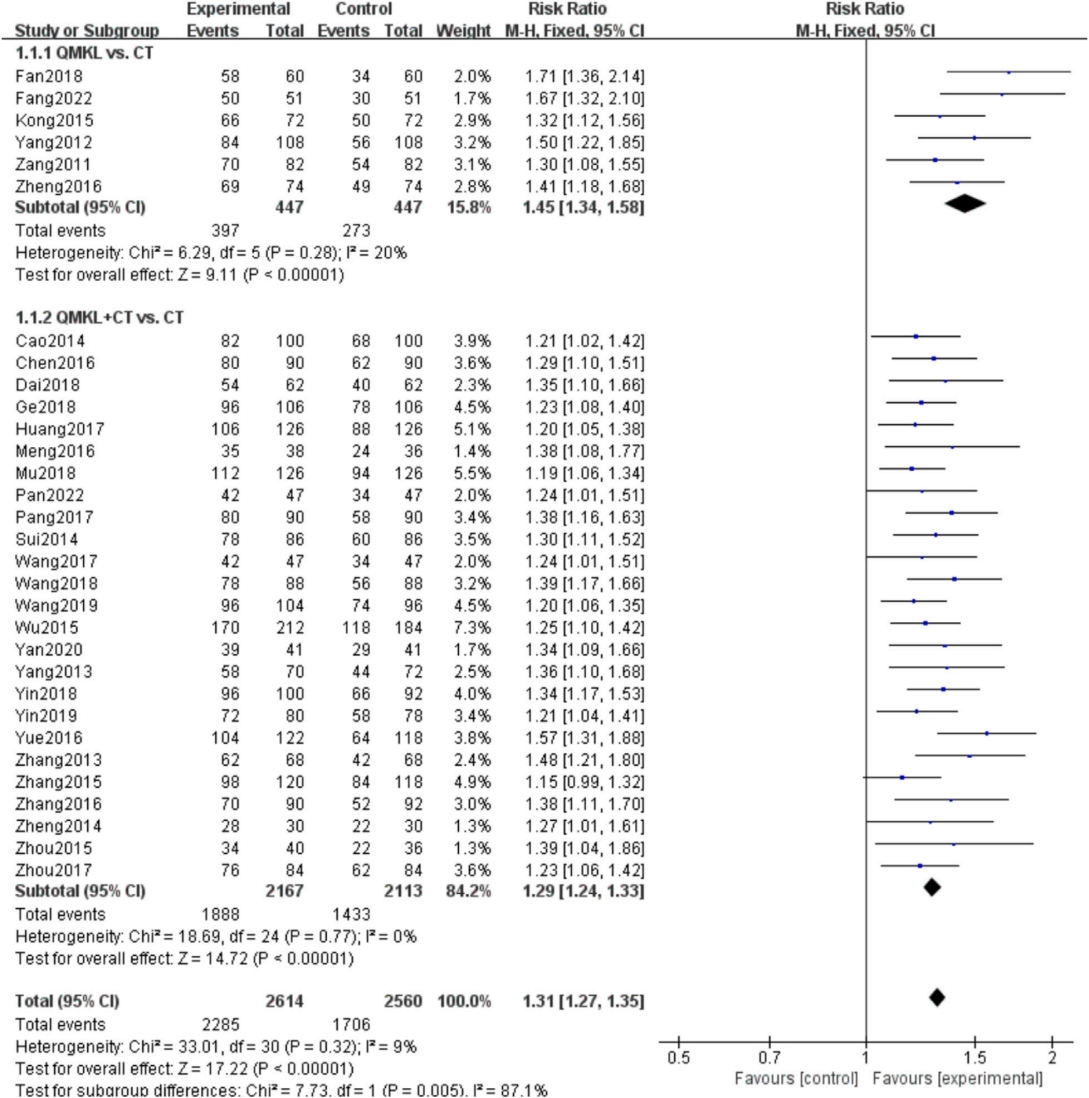
Meta-analysis results of the effect of QM vs. CT/QM plus CT on Overall efficacy.

A total of 31 RCTs (Fang et al., 2022; Fan et al., 2018; Zheng, 2016; Kong and Dan, 2015; Yang, 2012; Zang and Yang, 2011; Pan et al., 2022; Yan, 2020; Wang et al., 2019; Yin and Xiaohua, 2019; Ge, 2018; Dai et al., 2018; Mu et al., 2018; Wang, 2018; Yin, 2018; Pang, 2017; Zhou, 2017; Huang, 2017; Wang, 2017; Meng et al., 2016; Chen, 2016; Zhang et al., 2016; Yue, 2016; Zhou et al., 2015; Zhang, 2015; Wu et al., 2015; Sui et al., 2014; Zheng et al., 2014; Cao, 2014; Yang et al., 2013; Zhang, 2013) involving 2,859 participants and 5,175 eyes reported the overall response rate. Due to the low heterogeneity among the studies (*p* = 0.32, *I* ^*2*^ *=* 9%), a fixed-effects model was used for the meta-analysis. Subgroup analyses were performed based on the intervention (QM alone or QM plus CT). In the subgroup of QM vs. CT (6 RCTs (Fang et al., 2022; Fan et al., 2018; Zheng, 2016; Kong and Dan, 2015; Yang, 2012; Zang and Yang, 2011) involving 546 participants, 894 eyes), QM alone was superior to CT [RR = 1.45, 95% CI (1.34, 1.58), *p* < 0.00001], (Figure 4). In the QM plus CT subgroup (25 RCTs (Pan et al., 2022; Yan, 2020; Wang et al., 2019; Yin and Xiaohua, 2019; Ge, 2018; Dai et al., 2018; Mu et al., 2018; Wang, 2018; Yin, 2018; Pang, 2017; Zhou, 2017; Huang, 2017; Wang, 2017; Meng et al., 2016; Chen, 2016; Zhang et al., 2016; Yue, 2016; Zhou et al., 2015; Zhang, 2015; Wu et al., 2015; Sui et al., 2014; Zheng et al., 2014; Cao, 2014; Yang et al., 2013; Zhang, 2013) with 2,313 participants and 4,280 eyes), QM plus CT was superior to CT alone [RR = 1.29, 95% CI: (1.24, 1.33), *p* < 0.00001].

##### 3.4.1.2 Visual acuity

**FIGURE 5:**
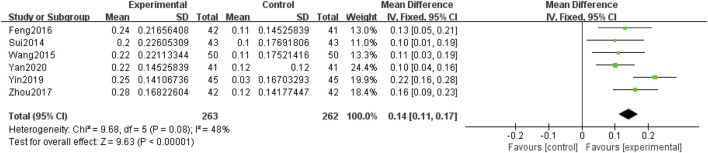
Meta-analysis results of the effect of QM plus CT on Visual acuity.

Seven included RCTs (Kong and Dan, 2015; Yan, 2020; Yin and Xiaohua, 2019; Wang et al., 2015; Zhou, 2017; Feng et al., 2016; Sui et al., 2014), involving 597 participants, reported on visual acuity. Subgroup analyses were performed based on the intervention (QM alone or QM plus CT). Because the subgroup of QM vs. CT involved only one study (1 RCT (Kong and Dan, 2015), with 72 participants), a descriptive analysis was employed. The results revealed that the experimental group exhibited better outcomes than the control group after treatment with QM, and QM alone was associated with improved visual acuity. In the subgroup of QM plus CT (6 RCTs (Yan, 2020; Yin and Xiaohua, 2019; Wang et al., 2015; Zhou, 2017; Feng et al., 2016; Sui et al., 2014), involving 525 participants), the fixed-effects model was used for meta-analysis due to the low heterogeneity among the studies (*p* = 0.08, *I* ^*2*^ = 48%). The effect of QM plus CT in improving visual acuity was better than that of CT alone [MD = 0.14, 95% CI (0.11, 0.17), *p* < 0.00001], (Figure 5).

##### 3.4.1.3 Retinal circulation time

**FIGURE 6:**
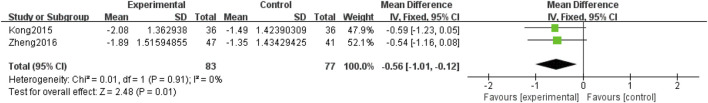
Meta-analysis results of the effect of QM vs. CT on Retinal circulation time.

Three included RCTs (Zheng, 2016; Kong and Dan, 2015; Ge, 2018), involving 266 participants, reported retinal circulation time. Subgroup analyses were performed based on the intervention (QM alone or QM plus CT). In the QM plus CT subgroup, a descriptive analysis was used because only one study was involved (1 RCT (Ge, 2018), with 106 participants). Notably, QM plus CT treatment was superior to CT alone. QM plus CT treatment can shorten the retinal circulation time. In the subgroup of QM vs. CT (2 RCTs (Zheng, 2016; Kong and Dan, 2015), 160 participants), a fixed-effects model was used for the meta-analysis due to low heterogeneity across studies (*p* = 0.91, *I* ^*2*^ = 0%). The results showed that QM alone resulted in a shorter retinal circulation time than CT [MD = −0.56, 95% CI (−1.01, −0.12), *p* = 0.01], (Figure 6).

##### 3.4.1.4 Macular thickness

**FIGURE 7:**
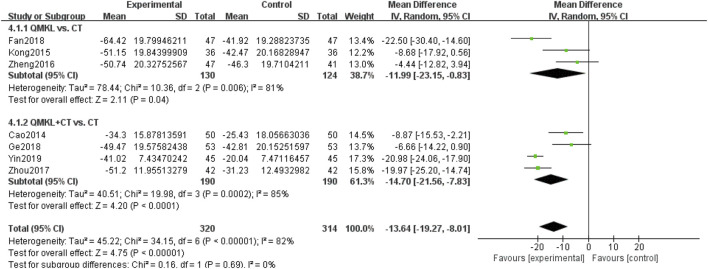
Meta-analysis results of the effect of QM vs. CT/QM plus CT on Macular thickness.

Seven included RCTs (Fan et al., 2018; Zheng, 2016; Kong and Dan, 2015; Yin and Xiaohua, 2019; Ge, 2018; Zhou, 2017; Cao, 2014), involving 634 participants, reported macular thickness. Due to the heterogeneity among the studies (*p* < 0.00001, *I* ^*2*^ = 82%), a random-effects model was used for the meta-analysis. Subgroup analyses were carried based on the intervention (QM alone or QM plus CT). In the subgroup of QM vs. CT (3 RCTs (Fan et al., 2018; Zheng, 2016; Kong and Dan, 2015), 254 participants), QM alone was more effective than CT in improving macular thickness [MD = −11.99, 95% CI (−23.15, −0.83), *p* = 0.04], (Figure 7). In the subgroup of QM plus CT (4 RCTs (Yin and Xiaohua, 2019; Ge, 2018; Zhou, 2017; Cao, 2014), 380 participants), QM plus CT was more effective in improving macular thickness compared to CT [MD = −14.70, 95% CI: (−21.56, −7.83), *p* < 0.0001).

#### 3.4.2 Secondary outcomes

The duration of diabetes is the primary risk factor for DR (Feng et al., 2016; American Diabetes Association, 2013). Diabetic patients are in a state of persistent hyperglycemia, and hyperglycemia is an important cause of microvascular complications (López-Contreras et al., 2020; Feng et al., 2016). Hyperglycemia leads to the accumulation of glycosylated toxic metabolites in blood vessels, resulting in pathological changes in retinal microvessels and blindness (Shaikh et al., 2021; Eynard and Repossi, 2019). Blood glucose levels serve as crucial indicators for understanding diabetes control, and they are also an important factor causing the occurrence of retinopathy (Wenbin et al., 2024; Feng et al., 2016). Additionally, metabolic diseases such as hyperglycemia and dyslipidemia are recognized risk factors for the development and progression of DR (Chou et al., 2020; Zhou et al., 2018; Yau et al., 2012; Mohamed et al., 2007). Lipid clearance leads to an increase in non-enzymatic oxidation and glycosylation, activates inflammation, and leads to vascular hyperpermeability and retinal barrier destruction (Hammer and Busik, 2017). Some studies (Yuan et al., 2020; Yanyu et al., 2023; Qiumei et al., 2003) have further confirmed that the TC is associated with diabetes, and the high expression level of the TC will influence the occurrence of diabetes mellitus. The TC level increases with the severity of the disease in the DR. The decrease in HDL-C level is positively correlated with DR, and HDL-C can cause DR (Benarous et al., 2011; Romero-Aroca et al., 2011). The increase in LDL-C levels is positively correlated with the development of DR (Benarous et al., 2011; Romero-Aroca et al., 2011). High concentrations of LDL-C have cytotoxic effects on vascular endothelial cells, which lead to the progression of DR (Murakami et al., 2021). Glycated hemoglobin is a widely used marker for blood glucose levels (Wenbin et al., 2024) and can also be used as a marker to screen for DR progression (Song et al., 2023). Therefore, the four indexes of blood lipid and glycosylated hemoglobin were used to reflect the condition of DR.

##### 3.4.2.1 TG

**FIGURE 8:**
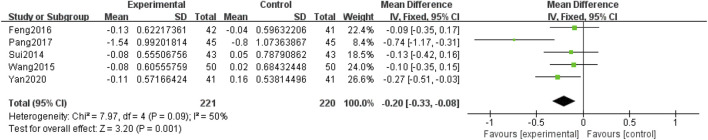
Meta-analysis results of the effect of QM plus CT on TG.

Five included RCTs (Yan, 2020; Pang, 2017; Feng et al., 2016; Wang et al., 2015; Sui et al., 2014) involving 441 participants, reported TG levels. Due to the low heterogeneity among the studies (*p* = 0.09, *I* ^*2*^ = 50%), a fixed-effects model was used for the meta-analysis. The meta-analysis compared QM plus CT. Notably, QM plus CT was superior to CT alone in improving TG levels [MD = −0.20, 95% CI (−0.33, −0.08), *p* = 0.001], (Figure 8).

##### 3.4.2.2 TC

**FIGURE 9:**
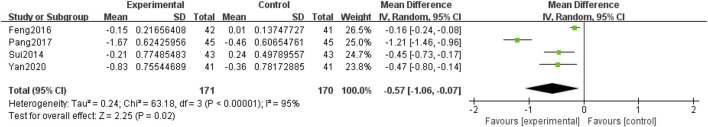
Meta-analysis results of the effect of QM plus CT on TC.

Four included RCTs (Yan, 2020; Pang, 2017; Feng et al., 2016; Sui et al., 2014), involving 341 participants, reported TC levels. Due to the heterogeneity among the studies (*p* < 0.0001, *I* ^*2*^ = 95%), a random-effects model was used for the meta-analysis. The meta-analysis compared QM plus CT. Notably, QM plus CT was superior to CT alone in improving TC levels [MD = −0.57, 95% CI (−1.06, −0.07), *p* = 0.02], (Figure 9).

##### 3.4.2.3 HDL-C

**FIGURE 10:**
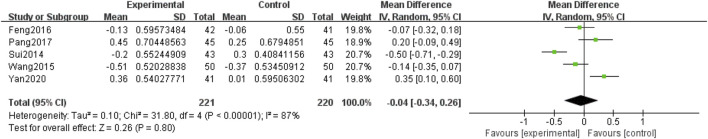
Meta-analysis results of the effect of QM plus CT HDL-C.

Five included RCTs (Yan, 2020; Pang, 2017; Feng et al., 2016; Wang et al., 2015; Sui et al., 2014), involving 441 participants, reported HDL-C levels. Due to the heterogeneity among the studies (*p* < 0.00001, *I* ^*2*^ = 87%), a random-effects model was used for the meta-analysis. The meta-analysis compared QM plus CT. Notably, no significant differences were observed between the two groups [MD = −0.04, 95% CI (−0.34, 0.26), *p* = 0.80], (Figure 10).

##### 3.4.2.4 LDL-C

**FIGURE 11:**
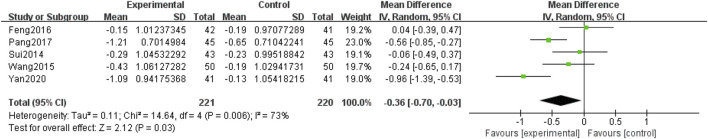
Meta-analysis results of the effect of QM plus CT on LDL-C.

Five included RCTs (Yan, 2020; Pang, 2017; Feng et al., 2016; Wang et al., 2015; Sui et al., 2014), involving 441 participants, reported LDL-C levels. Due to the heterogeneity among the studies (*p* = 0.006, *I* ^*2*^ = 73%), a random-effects model was used for the meta-analysis. The meta-analysis compared QM plus CT. The results showed that the difference between the two groups was statistically significant [MD = −0.36, 95% CI (−0.70, −0.03), *p* = 0.03], (Figure 11), and the QM plus CT reduced LDL-C levels better than CT.

##### 3.4.2.5 HbA1c

**FIGURE 12:**
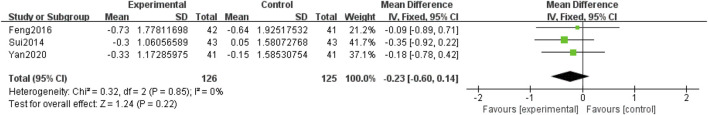
Meta-analysis results of the effect of QM plus CT on HbA1c.

Three included RCTs (Yan, 2020; Feng et al., 2016; Sui et al., 2014), involving 251 participants, reported HbA1c. Due to the low heterogeneity among the studies (*p* = 0.85, *I* ^*2*^ = 0%), a fixed-effects model was used for the meta-analysis. The meta-analysis compared QM plus CT. No significant differences were observed between the two groups [MD = −0.23, 95% CI (−0.60, 0.14), *p* = 0.22], (Figure 12).

### 3.5 Safety outcome

Adverse events were reported in 10 studies, of which 6 studies reported the specific occurrence of adverse events. Two studies Fang et al. (2022), Fan et al. (2018) involved treatment with QM vs. CT, while 8 studies (Yan, 2020; Wang et al., 2019; Dai et al., 2018; Yin, 2018; Wang, 2017; Wang et al., 2015; Sui et al., 2014; Cao, 2014) involved QM plus CT. The adverse events included a shadow fluttered before the eyes, gastrointestinal discomfort (included nausea, diarrhea, epigastric discomfort), proliferative diabetic retinopathy, renal injury, liver injury, and significant blood glucose fluctuations. Four studies reported no significant adverse events of QM, (Table 2).

**TABLE 2:** Occurrence of adverse events.

Study	Sample size	Adverse events		
	T	C	T	C
Wang et al. (2015)	50	50	0	0
Sui et al. (2014)	43	43	0	0
Cao (2014)	50	50	1 (a shadow fluttered before my eyes)	4 (2 a shadow fluttered before my eyes; 1 nausea and diarrhea; 1 significant blood glucose fluctuations)
Fang et al. (2022)	51	51	2 (1 proliferative diabetic retinopathy; 1 renal injury)	18 (7 proliferative diabetic retinopathy; 5 liver damage; 6 renal injury)
Fan et al. (2018)	47	47	2 (1 liver damage; 1 renal injury)	17 (4 proliferative diabetic retinopathy; 6 liver damage; 7 renal injury)
Wang et al. (2019)	52	48	0	0
Yan (2020)	41	41	2 (1 liver damage; 1 proliferative diabetic retinopathy)	8 (3 liver damage; 3 proliferative diabetic retinopathy; 2 renal injury)
Wang (2017)	47	47	0	2 (2 epigastric discomfort)
Yin (2018)	50	46	0	0
Dai et al. (2018)	31	31	1 (a shadow fluttered before my eyes)	6 (3 a shadow fluttered before my eyes; 2 diarrhea; 1 nausea)

Abbreviations:T, treatment group; C, control group.

In the QM vs. CT, three adverse events, namely, proliferative diabetic retinopathy, renal injury, liver injury, were involved. The meta-analysis results showed that the QM group alone had significantly fewer three adverse enents than the CT group [proliferative diabetic retinopathy, RR = 0.13, 95% CI (0.02, 0.70), *p = 0.02*]; renal injury, RR = 0.15, 95% CI: (0.04, 0.66), *p* = 0.01; liver damage, RR = 0.13, 95% CI (0.02, 0.70), *p* = 0.02], (Table 3).

**TABLE 3:** Meta-analysis results of the adverse events.

Adverse events	Different intervention measures	NO.S	RR	95%CI	I^2^	*p*-Value
A shadow fluttered before the eyes	QM vs. CT	—	—	—	—	—
	QM plus CT	2	0.40	[0.08, 2.00]	0%	0.26
Gastrointestinal discomfort^a^	QM vs. CT	—	—	—	—	—
	QM plus CT	3	0.20	[0.04, 1.13]	0%	0.07
Proliferative diabetic retinopathy	QM vs. CT	2	0.13	[0.02, 0.70]	0%	0.02
	QM plus CT	1	0.33	[0.04, 3.07]	—	0.33
Renal injury	QM vs. CT	2	0.15	[0.04, 0.66]	0%	0.01
	QM plus CT	1	0.20	[0.01, 4.04]	—	0.29
Liver damage	QM vs. CT	2	0.13	[0.02, 0.70]	0%	0.02
	QM plus CT	1	0.33	[0.04, 3.07]	—	0.33
Significant blood glucose fluctuations	QM vs CT	—	—	—	—	—
	QM plus CT	1	0.33	[0.01, 7.99]	—	0.50

Abbreviations: NO.S, numbers of studies; RR, risk ratio; CI, confidence interval; I^2^, heterogeneity; QM, QiMing granules; CT, conventional therapy;/not applicable.

^a^ Gastrointestinal discomfort included nausea, diarrhea, epigastric discomfort.

In the QM plus CT, a total of six types adverse events were invovled. The meta-analysis results showed that the two groups performed similarly for a shadow fluttered before the eyes, gastrointestinal discomfort, proliferative diabetic retinopathy, renal injury, liver injury, and significant blood glucose fluctuations (*p* > 0.05) (Table 3).

### 3.6 Sensitivity analyses

In the subgroup of QM vs. CT, the results of the sensitivity analysis indicated that the pooled results of overall efficacy and retinal circulation time were stable. In the QM plus CT subgroup, the results of the sensitivity analysis indicated that the pooled results of overall efficacy, visual acuity, macular thickness, TG, HDL-C, and HbA1c were stable. However, for the macular thickness outcome with QM vs. CT, the meta-analysis results changed after excluding one study (Kong and Dan, 2015), indicating a lack of robustness in the meta-analysis results. Different from other studies, the sample size in this study was less than 40 patients which may lead to the clinical heterogeneity. Similarly, for the TC outcome with QM plus CT, the meta-analysis results changed when two studies were excluded one by one (Sui et al., 2014; Yan, 2020), suggesting a lack of robustness in the meta-analysis results. Different from other studies, the duration of treatment in this study was less than 3 months, which may lead to the clinical heterogeneity. Additionally, for the LDL-C outcome with QM plus CT, the meta-analysis results changed when three studies were excluded one by one (Kong and Dan, 2015; Pang, 2017; Yan, 2020), further highlighting the lack of robustness in the meta-analysis results. Different from other studies, the duration of treatment in this study was less than 3 months, which may lead to the clinical heterogeneity. Details of the sensitivity analyses are provided in Supplementary Material S5. The meta-analysis of macular thickness, TC, and LDL-C were not stable, prompting caution in interpreting the results.

### 3.7 Subgroup analysis

Subgroup analyses were conducted for each efficacy outcome based on treatment time (≤3 m, > 3 m) for both interventions. For QM vs. CT, the results of these subgroup analyses were consistent with the overall results (Table 4). For QM plus CT, the interaction effect of macular thickness was significantly different under different treatment durations (≤3 m [MD = −17.14; 95% CI: (−23.61, −10.67); *I* ^*2*^ = 81%]; > 3 m [MD = −6.66; 95% CI: (−14.22, −0.90); *P*_interaction_ of duration = 0.04). The interaction effect of TC was significant (≤3 m [MD = −0.32; 95% CI: (−0.56, −0.08); *I* ^*2*^ = 70%; > 3 m [MD = −1.21, 95% CI: (−1.46, −0.96); *P*_interaction_ of duration <0.00001), (Table 5).

**TABLE 4:** Subgroup analysis of the outcomes of QM vs. CT.

Subgroup		NO.S	MD/RR	95%CI	*I* ^*2*^ (%)	*P* Interaction
Different treatment duration						
Overall efficacy	≤3 m	2	1.40	1.22 to 1.61	11	0.47
	>3 m	4	1.49	1.35 to 1.64	36	

Abbreviations: No.S, numbers of studies; MD, mean difference; RR, risk ratio; CI, confidence interval; *I* ^*2*^, heterogeneity; *P* interaction, *P* for interaction; QM, QiMing granules; CT, conventional therapy;/, not applicable.

**TABLE 5:** Subgroup analysis of the outcomes of QM plus CT.

Subgroup				NO.S		MD/RR		95%CI		*I* ^*2*^ (%)		*P*_interaction_	
Different treatment duration													
Overall efficacy		≤3 m		14		1.26		1.21 to 1.32		0		0.19	
		>3 m		11		1.32		1.25 to 1.39		18			
Visual acuity		≤3 m		5		0.14		0.10 to 0.19		56		0.47	
		>3 m		1		0.11		0.03 to 0.19		—			
Macular thickness		≤3 m		3		−17.14		−23.61 to −10.67		81		0.04	
		>3 m		1		−6.66		−14.22 to 0.90		—			
TG		≤3 m		3		−0.17		−0.32 to −0.02		0		0.48	
		>3 m		2		−0.27		−0.48 to −0.05		84			
TC		≤3 m		3		−0.32		−0.56 to −0.08		70		<0.00001	
		>3 m		1		−1.21		−1.46 to −0.96		—			
HDL-C		≤3 m		3		−0.08		−0.57 to 0.42		93		0.76	
		>3 m		2		0.02		−0.32 to 0.35		72			
LDL-C		≤3 m		3		−0.33		−0.95 to 0.30		84		0.76	
		>3 m		2		−0.43		−0.74 to −0.13		36			

Abbreviations: No.S, numbers of studies; MD, mean difference; RR, relative risk; CI, confidence interval; *I* ^*2*^, heterogeneity; *P* interaction, *P* for interaction; QM, QiMing granules; CT, conventional therapy; TG, triglyceride; TC, total cholesterol; HDL-C, High-density lipoprotein cholesterol; LDL-C, Low-density lipoprotein cholesterol;/, not applicable.

### 3.8 Risk of publication bias

**FIGURE 13:**
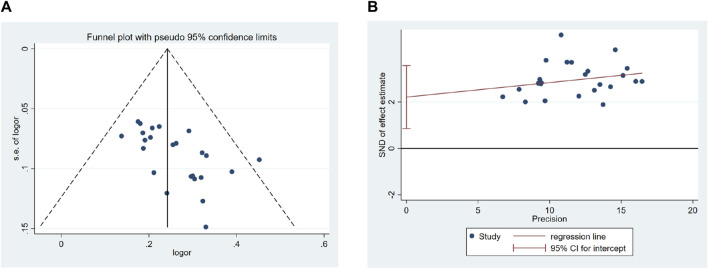
Effect of QM plus CT vs. CT on Overall efficacy. **(A)** Funnel plots revealed the publication bias. **(B)** Egger’s test quantified the publication bias.

We conducted a publication bias analysis for the overall efficacy of QM plus CT. Because the number of trials exceeded 10, we used both a funnel chart and Egger’s test to determine whether there was publication bias. The funnel plot (Figure 13A), generated using the total effective rate as an indicator, was not completely symmetrical, indicating a potential risk of publication bias. Furthermore, the Egger’s test show significant publication bias, with a *p*-value of 0.003 (Figure 13B).

### 3.9 Certainty of evidence

The GRADE method was used to systematically evaluate the certainty of nine outcome measures (Table 6). The results indicated a moderate quality of evidence for overall efficacy and TG levels. Additionally, the certainty of evidence for TC was very low. Finally, the certainty of evidence was low for the remaining visual acuity, retinal circulation time, macular thickness, HDL-C, LDL-C, and HbA1c. The main reasons for the degradation of evidence certainty may be related to the poor methodological certainty of the included RCTs, large heterogeneity among studies, small sample size, potential publication bias, and limited the number of included RCTs.

**TABLE 6:** Summary of the study findings.

NO.	Study design	Certainty assessment					Summary of results							Importan-ce
		Risk of bias	Inconsi-stency	Indirec-tness	Impreci-sion	Others	No of patients		Effect (95%)		Certainty			
							T	C	Relative	Absolute				
Overall efficacy (QM vs. CT/QM plus CT)														
31	RCT	not Serious	not Serious	not Serious	not Serious	Seriousa	1444 (2614^f^)	1415 (2560^f^)	RR = 1.31 (1.24, 1.33)	-	⊕⊕⊕○	Mod-erate		Critical
Visual acuity (QM vs. CT/QM plus CT)														
7	RCT	Seriousb	Seriousc	not Serious	not Serious	not Serious	299	298	-	MD = 0.15 (0.11, 0.19)	⊕⊕ ○○	Low	Critical	
Retinal circulation time (QM vs. CT/QM plus CT)														
3	RCT	not Serious	not Serious	not Serious	Seriousd	Seriouse	136	130	-	MD = −0.63 (−0.95, −0.30)	⊕⊕ ○○	Low	Critical	
Macular thickness (QM vs*.*CT/QM plus CT)														
7	RCT	Seriousb	Seriousc	not Serious	not Serious	not Serious	320	314	-	MD = −13.64 (−19.27, −8.01)	⊕⊕ ○○	Low	Critical	
TG (QM plus CT)														
5	RCT	not Serious	not Serious	not Serious	not Serious	Seriouse	221	220	-	MD = −0.20 (−0.33, −0.08)	⊕⊕⊕○	Mod-erate	Important	
TC (QM plus CT)														
4	RCT	not Serious	Seriousc	not Serious	Seriousd	Seriouse	171	170	-	MD = −0.57 (−1.06, −0.07)	⊕○ ○○	Very low	Important	
HDL-C (QM plus CT)														
5	RCT	not Serious	Seriousc	not Serious	not Serious	Seriouse	221	220	-	MD = −0.04 (−0.34, 0.26)	⊕⊕ ○○	Low	Important	
LDL-C (QM plus CT)														
5	RCT	not Serious	Seriousc	not Serious	not Serious	Seriouse	221	220	-	MD = −0.36 (−0.70, 0.03)	⊕⊕ ○○	Low	Important	
HbA1c (QM vs. CT/QM plus CT)														
3	RCT	not Serious	not Serious	not Serious	Seriousd	Seriouse	126	125	-	MD = −0.23 (−0.60,0.14)	⊕⊕ ○○	Low	Important	

Abbreviation: RCT, randomized controlled trial; MD, mean difference; RR, risk ratio; CI, confidence interval.

^a^ Downgrade by one level: There was a risk of publication bias.

^b^ More than 25% of the studies were those with a higher risk of overall bias.

^c^ Heterogeneity among the studies was fairly high.

^d^ The optimal information sample size was less than 400 participants.

^e^ The number of RCTs, was s less than 6.

^f^ The number of eyes involved.

## 4 Discussion

DR is a complication of diabetes characterized by damage to the retinal vascular barrier and hemodynamic changes (Yuzhi et al., 2019). Traditional Chinese medicine views, DR within the context of “wasting-and-thirst eye disease”, attributing its onset to imbalances in qi and blood, Yin and Yang, body fluid and qi machinery, and deficiency of viscera (Jing and Junping, 2024). Conversely, Western medicine relates DR’s pathological mechanisms to physiological processes such as hyperglycemia-induced expression of growth factors and cytokines, activation of oxidative stress and the polyol pathway, and disturbances in hemodynamics, leading to neuroinflammation and vascular dysfunction in the retina (Jampol et al., 2020; Kinuthia et al., 2020).

### 4.1 Summary of results

This systematic review included 33 RCTs involving 3,042 participants to evaluate the safety and efficacy of QM alone or in combination with CT in the treatment of DR. Primary outcome measures included overall efficacy, visual acuity, retinal circulation time, and macular thickness, while secondary outcomes included TG, TC, HDL-C, LDL-C, and HbA1c. Adverse events were used as safety indicators. The analysis revealed that QM significantly improved the overall efficacy and visual acuity, shortened retinal circulation time, and improved macular thickness, suggesting that QM can significantly improve the clinical symptoms and quality of life of patients with DR, whether used alone or in combination with CT. When used in combination with CT, QM demonstrated significant effects on blood lipid indexes, regulating TG and reducing TC and LDL-C levels, albeit with no significant impact on HDL-C and HbA1c. Moreover, based on the number of adverse events reported in the two groups, the experimental group had fewer adverse events. The results showed that the incidence of adverse events in the QM group was lower than that in the control group, especially in reducing the impairment of liver and kidney function and alleviating the progression of proliferative retinopathy, suggesting that QM was safer. The safety of QM was high in a randomized, double-blind,double-dummy multicenter trial (Junguo et al., 2006). Modern network pharmacology studies have found that *Pueraria lobata* and *Radix Astragali* have various pharmacological effects, including immunoregulatory, anti-inflammatory, anti-diabetic, and lipid level-reducing effects (Dong, 2016; Guo et al., 2017; Lee et al., 2019; Liangzhe et al., 2017; Ny et al., 2021; Yin et al., 2014). Puerarin inhibits the expression of vascular endothelial growth factor (VEGF) (Jintao et al., 2018), while Lycium wolfberry exerts an anti-inflammatory effect (Baozhou, 2022). A study (Hangzhu et al., 2022) have screened 33 active ingredients present in QM and 59 targets for the treatment of DR. QM exerts its therapeutic effects in the treatment of DR by targeting multiple signaling pathways implicated in the pathogenesis of the condition. Specifically, QM intervenes in the AGE-RAGE signaling pathway in diabetic complications, as well as in Type I and Type II diabetes mellitus. Additionally, it modulates VEGF and transforminggrowthfactor-β (TGF-β) signaling pathways, among others, to effectively treat DR. Another study (Ruyu et al., 2023) also identified the core target of QM, suggesting its efficacy in the treatment of DR. Animal experiments (Hejiang et al., 2011; Xiyu et al., 2020; Yanjie et al., 2021; Zhongmei et al., 2023) have corroborated these findings by demonstrating QM’s ability to reduce the thickness of the retinal capillary basement membrane and enhance visual function. This protective effect may be because flavonol compounds, hirudin, and other components of QM, which protect retinal ganglion cells from high hyperglycemia-induced damage by promoting extracellular regulated kinase and angiogenesis inhibitor protein 1 signaling, thereby protecting the retina and vision (Xiangwu and Zhenshun, 2008; Zhao et al., 2020; Zhaoyang et al., 2021). QM also regulates the tumor necrosis factor (TNF), vascular endothelial growth factor A (VEGFA), and nuclear factor kappa nuclear factor kappa-B (NF-kB) signaling pathways, which play crucial roles in regulating retinal vascular cell apoptosis and maintaining the blood-retinal barrier, thereby reducing the appearance of dark shadows (Aveleira et al., 2010; Thomas et al., 2017; Zhaoyang et al., 2021). QM demonstrates efficacy in improving clinical symptoms, enhancing visual acuity, optimizing blood lipid levels, and mitigating adverse reactions in DR patients. Its multifaceted mechanism of action and safety profile support its role as a promising therapeutic option for DR management.

### 4.2 Subgroup and sensitivity analysis

Statistical heterogeneity observed among studies may stem from various clinical or methodological factors (Melsen et al., 2014). To determine the influence of other factors on the efficacy of QM, subgroup analyses were conducted, considering different treatment durations. Based on the results from different treatment time subgroups, it was observed that there are differences in the improvement of macular thickness and reduction of TC level. Specifically, when the treatment time is ≤ 3m, QM plus CT has a better effect on improving macular thickness, and QM plus CT has better results in reducing TC levels. However, it is important to note that further studies are required to validate these conclusions and ensure their reliability.

Additionally, the sensitivity analyses showed that the meta-analysis results were not affected by removing any individual study, indicating the robustness of the overall efficacy (QM vs. CT), retinal circulation time (QM vs. CT), overall efficacy (QM plus CT), Visual acuity (QM plus CT), Macular thickness (QM plus CT), TG (QM plus CT), HDL-C (QM plus CT), HbA1c (QM plus CT). However, the sensitivity analysis demonstrated that the meta-analysis results were not robust to macular thickness (QM vs. CT), TC, and LDL-C (QM plus CT). Read the original article to consider unstable reasons, which may be related to the small number of included studies, the small sample size, and the differences in control dosage of QM, which may be a source of heterogeneity. Furthermore, due to the studies published in Chinese, which may result in language bias. Finally, the intervention measures, may be is one of the reasons lead to the result is not stable.

### 4.3 Risk of bias and certainty of evidence

Despite our efforts to minimize bias in the study, certain limitations were inevitable. Randomization and blinding were not reported in most studies, making it challenging to assess the risks of selection and performance bias. Additionally, none of the included studies reported trial registration, leading to a lack of transparency in the study process. As a result, 75.80% of the studies were classified as having some concern for overall bias, with 24.20% considered at high risk, thereby reducing the credibility of the results. Therefore, the results of this review should be considered with caution.

The GRADE approach was used to assess the certainty of the evidence in this review. The overall efficacy and TG were moderate; visual acuity, retinal circulation time, macular thickness, HDL-C, LDL-C, and HbA1c were low; and TC was very low. The certainty of TC was considered low, prompting caution in interpreting the results. The primary reasons for downgrading included inconsistency and others. High-quality, large-sample, and multi-center RCTs should be carried out to improve the certainty of the evidence for QM in DR.

### 4.4 Advantages and limitations

Previous systematic reviews have revealed that QM is effective and safe for the treatment of DR. However, this study has some advantages over previous studies. Firstly, 33 RCTs were included in this study, which is the largest number of included studies to date. Secondly, this study comprehensively evaluated the efficacy of QM in the treatment of DR by evaluating nine outcome measures. Thirdly, we performed subgroup analyses based on treatment duration to explore the influence of certain characteristics on treatment response. Fourth, sensitivity analysis and GRADE evaluation were conducted in this study, adhering strictly to reporting standards.

However, this study still has some limitations. Firstly, this study did not search gray literature, and there may be a certain degree of missed detection. Secondly, all the retrieved literature was in Chinese, and there was no English literature, which may lead to bias. Thirdly, the number and sample size of the included studies were small. Fourth, when the number of patients was reported but the number of diseased eyes was not reported in the article, the default number of diseased eyes was two, which may also cause a certain bias. Fifth, the included studies had a risk of bias that reduced the credibility of the evidence. The sensitivity analysis revealed heterogeneity, and the results were not robust, indicating reduced credibility.

### 4.5 Suggestions

Based on the conclusions and limitations of this study, some useful and feasible suggestions were proposed for future research. First, since no exact safety conclusions were found in this study, the monitoring and recording of adverse events should be standardized in future studies. Second, the effects of QM treatment duration need to be further studied. Finally, improve the quality of clinical research design, the reasonable standard of clinical research design: (a) clear the selection criteria of the research object, including the inclusion criteria and exclusion criteria. (b) Adequate sample size was designed and the appropriate sample size was determined by selecting the correct sample size calculation formula according to the different outcome indicators. (c) Multidimensional consideration of the design of interventions. Including clear the dosage, course of treatment, the manufacturer, batch number. The acceptability of subjects and the extensibility of interventions were considered when necessary. The control group should be compared to placebo, blank is advisable. (d) select authenticity and reliability are good indicators, attaches great importance to the science of indicators. (e) to carry out with the correct method randomized, double-blind, multicenter, large sample and long-term follow-up studies, and strictly follow the standard (CONSORT) clinical trial report. At the same time, the correct allocation concealment method is used to improve the credibility of the test results.

## 5 Conclusion

This study confirmed that QM, whether used alone or in combination, can improve the overall efficacy, enhance visual acuity, improve macular thickness, shorten retinal circulation time, and reduce the levels of TG, TC, and LDL-C in DR patients in an all-round and multi-channel manner. However, due to the low quality of the evidence in the included studies, the overall level of evidence was not high. More high-quality, multi-center, and large-sample studies are still needed to confirm the results.

## Data Availability

The original contributions presented in the study are included in the article/Supplementary Material, further inquiries can be directed to the corresponding authors.
