# Supplementary material for: QiMing granules for diabetic retinopathy: a systematic review and meta-analysis of randomized controlled trials
Source: Front Pharmacol. 2024 Aug 22;15:1429071. doi: 10.3389/fphar.2024.1429071 (PMC11374745; doi:10.3389/fphar.2024.1429071)
Supplement: Supplementary file 3 [file DataSheet1.pdf]

Supplementary Material

1. Detailed information for Qiming granules

QiMing granules (Zhejiang Sansheng Mandi Pharmaceutical Co., LTD., Zhejiang China, SFDA approval number Z20090036) is an oral Chinese patent medicine, which was approved by the State Food and Drug Administration in 2009 as the first new traditional Chinese medicine drug for the treatment of diabetic retinopathy (2024a; 2024b). It is a new proprietary Chinese medicine formulation developed by Professors Liao Pinzheng and Duan Junguo's team at Chengdu University of Traditional Chinese Medicine (Xiyu et al., 2020), its contains eight medicinal herbs: Astragalus aaronii [Fabaceae/Leguminosae],Pueraria montana var. Lobata [Fabaceae/Leguminosae], Rehmannia glutinosa [Scrophulariaceae], Lycium chinense[Solanaceae Juss.], Cassia obtusifolia L. [Fabaceae/Leguminosae], Leonurus japonicus Houtt. [Labiatae/Lamiaceae], Typha latifolia L. [Typhaceae], and Whitmania pigra Whitma [Hirudo] (Hai Jie, 2013).Table 1 presents the chemical composition, Chinese names, and medicinal parts used of the QM.

TABLE 1 | Raw materials of QM.

Name	Chemical Composition	Chinese Name	Medicinal Parts
Astragalus aaronii	Flavonoid, Saponins, Polysaccharides	Huang Qi	Root
Pueraria montana var. lobata	Puerarin, daidzin, daidzein, and formononetin	Ge Gen	Root
Rehmannia glutinosa	Cycloalkene ether terpenoids, ionone, phenylethyl alcohol glycosides, triterpene, flavonoids, and sugar	Di Huang	Root
Lycium chinense	Sugars, sterols, flavonoids, alkaloids	Gou Qizi	Fruit
Cassia obtusifolia L.	Anthraquinone compounds, naphthalene and pyranoid ketone	Jue Mingzi	Seed
Leonurus japonicus Houtt.	Alkaloids, flavonoids, fatty acids, phenylpropanol glycosides, diterpenoids, volatile oils	Chong Weizi	Fruit
Typha latifolia L.	Naringenin, isorhamnetin, quercetin	Pu Huang	Pollen
Hirudo	Multiple enzymes, Peptides, alkaloids, carbohydrates, lipids, amino acids, trace elements	Shui Zhi	Whole animal

Abbreviation: QM: QiMing granules; *, *Whitmania pigra Whitman* is animal medicine.

All botanical drugs in QM were fully validated using http://www.plantsoftheworldonline.org.

References

2024a. Our eye team research and development of innovative TCM stilbene particles, Ming Dan red blood oral liquid comprehensive news listed in the latest national health insurance directory _ _ chengdu university of traditional Chinese medicine. Available at: https://www.cdutcm.edu.cn/ (accessed 2024/6/22 2024).

2024b. National Medical Products Administration data query. Available at: https://www.nmpa.gov.cn/datasearch/search-info.html?nmpa=aWQ9MzQ0NDY4MDFhZWUxNDE3ZmM2ZGU0N2Q0NDBjOTk1ZDQmaXRlbUlkPWZmODA4MDgxODNjYWQ3NTAwMTg0MDg4MWY4NDgxNzlm (accessed 2024/6/22 2024).

Hai Jie, Z. (2013). Research and application of Qiming granules in the treatment of diabetic retinopathy. Journal of Modern Medicine & Health. 29(07), 1030-1032

Xiyu, J., Hejiang, Y., and Yulin, Q. (2020). Discussion on the treatment of diabetic retinopathy with Qiming Granules based on the theory of five wheels and eight regions. China Journal of Traditional Chinese Medicine. 35(05), 2494-2496.

2. Search Queries

The search queries for China National Knowledge Infrastructure Database (CNKI), Wanfang Database (Wanfang), VIP Database for Chinese Technical Periodicals (VIP), Chinese Biomedical Literature Database (SinoMed), Web of Science, PubMed, Embase, and Cochrane Library are shown in Table 2.

Table 2 Search queries for each database.

Database	Search query
CNKI<br><br>(Searched from inception to Mar. 27, 2024 and found 81 literature)	SU %= '糖尿病视网膜病变' + '糖网病' + '糖尿病性视网膜病变' AND SU %= '芪明颗粒' AND SU %= '随机' + '临床' + '疗效观察' + '随机对照试验' + '临床试验' + '临床观察' + '临床研究' + '中西医结合' AND FT = '随机' NOT (SU %= '综述' + '系统评价' + 'Meta 分析' + '研究进展' + '荟萃分析' + '网状 Meta 分析' AND TI = '兔' + '鼠' + '蛙' + [illegible]
Wanfang<br><br>(Searched from inception to Mar. 27, 2024 and found 36 literature)	((((主题=糖尿病视网膜病变 OR 糖网病 OR 糖尿病性视网膜病变) AND 题名=芪明颗粒) AND 题名=(随机 OR 临床 OR 疗效观察 OR 随机对照试验 OR 临床试验 OR 临床观察 OR 临床研究 OR 中西医结合)) AND 随机) NOT 题名=(综述 OR 系统评价 OR Meta 分析 OR 研究进展 OR 荟萃分析 OR 网状 Meta 分析
VIP<br><br>(Searched from inception to Mar. 27, 2024 and found 30 literature)	M=(糖尿病视网膜病变 OR 糖网病 OR 糖尿病性视网膜病变) AND M=(芪明颗粒) AND M=(随机 OR 临床 OR 疗效观察 OR 随机对照试验 OR 临床试验 OR 临床观察 OR 临床研究 OR 中西医结合) AND (U=随机) NOT M=(综述 OR 系统评价 OR Meta 分析 OR 研究进展 OR 荟萃分析 OR 网状 Meta 分析 OR 兔 OR

SinoMed<br><br>(Searched from inception to Mar. 27, 2024 and found 59 literature)	((("糖尿病视网膜病变"[常用字段:智能] OR "糖网病"[常用字段:智能] OR "糖尿病性视网膜病变"[常用字段:智能]) AND "芪明颗粒"[常用字段:智能] AND("随机"[常用字段:智能] OR "临床"[常用字段:智能] OR "疗效观察"[常用字段:智能] OR "随机对照试验"[常用字段:智能] OR "临床试验"[常用字段:智能] OR "临床观察"[常用字段:智能] OR "临床研究"[常用字段:智能] OR "中西医结合"[常用字段:智能]) AND "随机"[全部字段:智能]) NOT("综述"[标题:智能] OR "系统评价"[标题:智能] OR "Meta 分析"[标题:智能] OR "研究进展"[标题:智能] OR "荟萃分析"[标题:智能] OR "网状 Meta 分析"[标题:智能] OR " 兔"[标题:智能] OR "鼠"[标题:智能] OR "蛙"[标
Web of Science<br><br>(Searched from inception to Mar. 27, 2024 and found 8 literature)	#1 TS=(Randomized Controlled Trial OR Clinical Trial OR Randomized Controlled Trials OR randomized controlled study OR randomized controlled trial OR randomized study OR randomized trial OR randomized placebo-controlled study OR randomized placebo-controlled trial OR randomized placebo controlled OR randomized placebo-controlled OR randomized double-blind OR randomized double blind)<br><br>#2 TS=(Diabetic retinopathy OR DR OR Diabetic Retinopathies OR Retinopathies, Diabetic OR Retinopathy, Diabetic OR diabetes retinopathy OR diabetic retinopathy)<br><br>#3 TS=(Qiming Keli OR qiming granules)<br><br>#4 #1 AND #2 AND #3

PubMed<br><br>(Searched from inception to Mar. 27, 2024 and found 1 literature)	#1 ((((((((((((Randomized Controlled Trial[Publication Type]) OR (Randomized Controlled Trials[MeSH Terms])) OR (Clinical Trial[Publication Type])) OR (randomized controlled study[Title/Abstract])) OR (randomized controlled trial[Title/Abstract])) OR (randomized study[Title/Abstract])) OR (randomized trial[Title/Abstract])) OR (randomized placebo-controlled study[Title/Abstract])) OR (randomized placebo-controlled trial[Title/Abstract])) OR (randomized placebo controlled[Title/Abstract])) OR (randomized placebo-controlled[Title/Abstract])) OR (randomized double-blind[Title/Abstract])) OR (randomized double blind[Title/Abstract])<br><br>#2 ((((((Diabetic retinopathy[Title/Abstract]) OR (DR[Title/Abstract])) OR (Diabetic Retinopathies[Title/Abstract])) OR (Retinopathies, Diabetic[Title/Abstract])) OR (Retinopathy, Diabetic[Title/Abstract])) OR (diabetes retinopathy[Title/Abstract])) OR (diabetic retinopathy[Title/Abstract])<br><br>#3 ((((((Diabetic retinopathy[MeSH Terms]) OR (DR[MeSH Terms])) OR (Diabetic Retinopathies[MeSH Terms])) OR (Retinopathies, Diabetic[MeSH Terms])) OR (Retinopathy, Diabetic[MeSH Terms])) OR (diabetes retinopathy[MeSH Terms])) OR (diabetic retinopathy[MeSH Terms])<br><br>#4 (Qiming Keli[Title/Abstract]) OR (qiming granules[Title/Abstract])<br><br>#5 (qiming granules[MeSH Terms]) OR (Qiming Keli[MeSH Terms])<br><br>#6 #2 OR #3<br><br>#7 #4 OR #5<br><br>#8 #1 AND #6 AND #7

Embase<br><br>(Searched from inception to Mar. 27, 2024 and found 0 literature)	#1 dr:ab,ti OR 'diabetic retinopathies':ab,ti OR 'retinopathies, diabetic':ab,ti OR 'retinopathy, diabetic':ab,ti OR 'diabetes retinopathy'/exp OR 'diabetes retinopathy':ab,ti OR 'diabetic retinopathy'/exp OR 'diabetic retinopathy':ab,ti<br><br>#2 'qiming keli':ab,ti OR 'qiming granules':ab,ti<br><br>#3 'randomized controlled trials'/exp OR 'randomized controlled study':ab,ti OR 'randomized controlled trial':ab,ti OR 'randomized study':ab,ti OR 'randomized placebo-controlled study':ab,ti OR 'randomized placebo-controlled trial':ab,ti OR 'randomized placebo controlled':ab,ti OR 'randomized placebo-controlled':ab,ti OR 'randomized double-blind':ab,ti OR 'randomized double blind':ab,ti<br><br>#4 #1 AND #2 AND #3

Cochrane Library<br><br>(Searched from inception to Mar. 27, 2024 and found 2 literature)	#1 (Diabetic retinopathy):ti,ab,kw OR (DR):ti,ab,kw OR (Diabetic Retinopathies):ti,ab,kw OR (Retinopathies, Diabetic):ti,ab,kw OR (Retinopathies, Diabetic):ti,ab,kw OR (Retinopathies, Diabetic):ti,ab,kw OR (diabetic retinopathy):ti,ab,kw<br><br>#2 (Qiming Keli):ti,ab,kw OR (qiming granules):ti,ab,kw<br><br>#3 (randomized controlled study):ti,ab,kw OR (randomized controlled trial randomized study):ti,ab,kw OR (randomized trial):ti,ab,kw OR (randomized placebo-controlled study):ti,ab,kw OR (randomized placebo-controlled study):ti,ab,kw OR (randomized placebo controlled):ti,ab,kw OR (randomized placebo-controlled):ti,ab,kw OR (randomized double-blind):ti,ab,kw OR (randomized double blind):ti,ab,kw<br><br>#4 #1 AND #2 AND #3

Abbreviation: CNKI, China National Knowledge Infrastructure Database; Wanfang, Wanfang Database; VIP, VIP Database for Chinese Technical Periodicals; SinoMed, Chinese Biomedical Literature Database.

3. Overall efficacy criteria:

Drawn up in accordance with the state administration of traditional Chinese medicine's disease diagnosis and curative effect standard:

Remarkable effect: (1) visual acuity improved by more than 4 lines; (2) the number of microvascular hemangioma in the fundus (+ + + > > (+ +) or (+ +) - (+) or (+) - (-); fundus hemorrhage (+++)→(+) or (++)→(-); exudation (+++)→(++) or (++)-+) or (+)-(-). Fundus fluorescein angiography showed that the mean circulation time of the retina was significantly shortened when more than two indicators met the requirements. The macular edema was significantly reduced, the non-perfusion area was reduced, the leakage was significantly reduced, and more than 2 indicators met the requirements, and the degree of change was more than 20%.

Effective: (1) visual acuity improved more than 2 lines; (2) the number of fundus microhemangioma decreased; decreased fundus hemorrhage; there was a reduction in exudation; (3) Fundus fluorescein angiography showed that the mean retinal circulation time was shortened; macular edema was reduced; the non-perfusion area decreased; the leakage was significantly reduced; more than one index met the requirements; and the degree of change was more than 10%.

Invalid: the indicators did not reach the above effective standards.

Deterioration: retinal neovascularization and other proliferative changes; aggravated macular edema; the non-perfusion area was enlarged and the leakage increased.

4. The information on QM from the original literature

The information for each original literature on QM is presented in Table 3.

Table3 The information on QM from the original literature.

Study	Formulation	Source	Species	Quality control reported? (Y/N)	Chemical analysis reported? (Y/N)
Zheng 2016	QiMing granule	Zhejiang Wansheng Pharmaceutical Co.,Ltd*	Astragalus aaronii [Fabaceae/Leguminosae] Pueraria montana var. Lobata [Fabaceae/Leguminosae] Rehmannia glutinosa [Scrophulariaceae] Lycium chinense	N	N

			[Solanaceae Juss.]<br>Cassia obtusifolia L.<br>[Fabaceae/Leguminosae]<br>Leonurus japonicus Houtt.<br>[Labiatae/Lamiaceae]<br>Typha latifolia L.<br>[Typhaceae]<br>Whitmania pigra Whitman<br>[Hirudo]		
Chen 2016	QiMing granule	Zhejiang Wansheng Pharmaceutical Co.,Ltd	Astragalus aaronii<br>[Fabaceae/Leguminosae]<br>Pueraria montana var. Lobata<br>[Fabaceae/Leguminosae]<br>Rehmannia glutinosa	N	N

			[Scrophulariaceae]<br>Lycium chinense<br>[Solanaceae Juss.]<br>Cassia obtusifolia L.<br>[Fabaceae/Leguminosae]<br>Leonurus japonicus Houtt.<br>[Labiatae/Lamiaceae]<br>Typha latifolia L.<br>[Typhaceae]<br>Whitmania pigra Whitman<br>[Hirudo]		
Wang 2015	QiMing granule	Zhejiang Wansheng Pharmaceutical Co.,Ltd	Astragalus aaronii<br>[Fabaceae/Leguminosae]<br>Pueraria montana var. Lobata	N	N

[Fabaceae/Leguminosae]

Rehmannia glutinosa

[Scrophulariaceae]

Lycium chinense

[Solanaceae Juss.]

Cassia obtusifolia L.

[Fabaceae/Leguminosae]

Leonurus japonicus Houtt.

[Labiatae/Lamiaceae]

Typha latifolia L.

[Typhaceae]

Whitmania pigra Whitman

[Hirudo]

Sui 2014	QiMing granule	Zhejiang Wanma Pharmaceutical Co., Ltd.*	Astragalus aaronii [Fabaceae/Leguminosae]<br>Pueraria montana var. Lobata [Fabaceae/Leguminosae]<br>Rehmannia glutinosa [Scrophulariaceae]<br>Lycium chinense [Solanaceae Juss.]<br>Cassia obtusifolia L. [Fabaceae/Leguminosae]<br>Leonurus japonicus Houtt. [Labiatae/Lamiaceae]<br>Typha latifolia L. [Typhaceae]	N	N

Huang 2017	QiMing granule	/	Whitmania pigra Whitman [Hirudo] Astragalus aaronii [Fabaceae/Leguminosae] Pueraria montana var. Lobata [Fabaceae/Leguminosae] Rehmannia glutinosa [Scrophulariaceae] Lycium chinense [Solanaceae Juss.] Cassia obtusifolia L. [Fabaceae/Leguminosae] Leonurus japonicus Houtt. [Labiatae/Lamiaceae]	N	N

Yin 2019	QiMing granule	Zhejiang Wansheng Pharmaceutical Co.,Ltd	Typha latifolia L. [Typhaceae] Whitmania pigra Whitman [Hirudo] Astragalus aaronii [Fabaceae/Leguminosae] Pueraria montana var. Lobata [Fabaceae/Leguminosae] Rehmannia glutinosa [Scrophulariaceae] Lycium chinense [Solanaceae Juss.] Cassia obtusifolia L. [Fabaceae/Leguminosae]	N	N

			Leonurus japonicus Houtt.<br>[Labiatae/Lamiaceae]<br>Typha latifolia L.<br>[Typhaceae]<br>Whitmania pigra Whitman<br>[Hirudo]		
Zhou 2017	QiMing granule	Zhejiang Wansheng Pharmaceutical Co.,Ltd	Astragalus aaronii<br>[Fabaceae/Leguminosae]<br>Pueraria montana var. Lobata<br>[Fabaceae/Leguminosae]<br>Rehmannia glutinosa<br>[Scrophulariaceae]<br>Lycium chinense<br>[Solanaceae Juss.]	N	N

			Cassia obtusifolia L.<br>[Fabaceae/Leguminosae]<br>Leonurus japonicus Houtt.<br>[Labiatae/Lamiaceae]<br>Typha latifolia L.<br>[Typhaceae]<br>Whitmania pigra Whitman<br>[Hirudo]		
Cao 2014	QiMing granule	Zhejiang Wansheng Pharmaceutical Co.,Ltd	Astragalus aaronii<br>[Fabaceae/Leguminosae]<br>Pueraria montana var. Lobata<br>[Fabaceae/Leguminosae]<br>Rehmannia glutinosa<br>[Scrophulariaceae]	N	N

			Lycium chinense [Solanaceae Juss.] Cassia obtusifolia L. [Fabaceae/Leguminosae] Leonurus japonicus Houtt. [Labiatae/Lamiaceae] Typha latifolia L. [Typhaceae] Whitmania pigra Whitman [Hirudo]		
Zhang 2015	QiMing granule	Zhejiang Wanma Pharmaceutical Co., Ltd.	Astragalus aaronii [Fabaceae/Leguminosae] Pueraria montana var. Lobata [Fabaceae/Leguminosae]	N	N

			Rehmannia glutinosa<br>[Scrophulariaceae]<br>Lycium chinense<br>[Solanaceae Juss.]<br>Cassia obtusifolia L.<br>[Fabaceae/Leguminosae]<br>Leonurus japonicus Houtt.<br>[Labiatae/Lamiaceae]<br>Typha latifolia L.<br>[Typhaceae]<br>Whitmania pigra Whitman<br>[Hirudo]		
Zhang 2013	QiMing granule	Zhejiang Wanma Pharmaceutical Co.,	Astragalus aaronii	N	N

Ltd.	[Fabaceae/Leguminosae]
	Pueraria montana var. Lobata
	[Fabaceae/Leguminosae]
	Rehmannia glutinosa
	[Scrophulariaceae]
	Lycium chinense
	[Solanaceae Juss.]
	Cassia obtusifolia L.
	[Fabaceae/Leguminosae]
	Leonurus japonicus Houtt.
	[Labiatae/Lamiaceae]
	Typha latifolia L.
	[Typhaceae]
	Whitmania pigra Whitman

			[Hirudo]		
Yang 2013	QiMing granule	/	Astragalus aaronii<br>Astragalus aaronii<br>[Fabaceae/Leguminosae]<br>Pueraria montana var. Lobata<br>[Fabaceae/Leguminosae]<br>Rehmannia glutinosa<br>[Scrophulariaceae]<br>Lycium chinense<br>[Solanaceae Juss.]<br>Cassia obtusifolia L.<br>[Fabaceae/Leguminosae]<br>Leonurus japonicus Houtt.<br>[Labiatae/Lamiaceae]	N	N

Kong 2015	QiMing granule	Zhejiang Wanma Pharmaceutical Co., Ltd.	Typha latifolia L. [Typhaceae]<br>Whitmania pigra Whitman [Hirudo]<br>Astragalus aaronii [Fabaceae/Leguminosae]<br>Pueraria montana var. Lobata [Fabaceae/Leguminosae]<br>Rehmannia glutinosa [Scrophulariaceae]<br>Lycium chinense [Solanaceae Juss.]<br>Cassia obtusifolia L. [Fabaceae/Leguminosae]	N	N

			Leonurus japonicus Houtt.<br>[Labiatae/Lamiaceae]<br>Typha latifolia L.<br>[Typhaceae]<br>Whitmania pigra Whitman<br>[Hirudo]		
Zang 2011	QiMing granule	Zhejiang Wanma Pharmaceutical Co., Ltd.	Astragalus aaronii<br>[Fabaceae/Leguminosae]<br>Pueraria montana var. Lobata<br>[Fabaceae/Leguminosae]<br>Rehmannia glutinosa<br>[Scrophulariaceae]<br>Lycium chinense<br>[Solanaceae Juss.]	N	N

			Cassia obtusifolia L.<br>[Fabaceae/Leguminosae]<br>Leonurus japonicus Houtt.<br>[Labiatae/Lamiaceae]<br>Typha latifolia L.<br>[Typhaceae]<br>Whitmania pigra Whitman<br>[Hirudo]		
Yue 2016	QiMing granule	/	Astragalus aaronii<br>[Fabaceae/Leguminosae]<br>Pueraria montana var. Lobata<br>[Fabaceae/Leguminosae]<br>Rehmannia glutinosa<br>[Scrophulariaceae]	N	N

			Lycium chinense<br>[Solanaceae Juss.]<br>Cassia obtusifolia L.<br>[Fabaceae/Leguminosae]<br>Leonurus japonicus Houtt.<br>[Labiatae/Lamiaceae]<br>Typha latifolia L.<br>[Typhaceae]<br>Whitmania pigra Whitman<br>[Hirudo]		
Fang 2022	QiMing granule	Zhejiang Wansheng Pharmaceutical Co.,Ltd	Astragalus aaronii<br>[Fabaceae/Leguminosae]<br>Pueraria montana var. Lobata<br>[Fabaceae/Leguminosae]	N	N

			Rehmannia glutinosa [Scrophulariaceae] Lycium chinense [Solanaceae Juss.] Cassia obtusifolia L. [Fabaceae/Leguminosae] Leonurus japonicus Houtt. [Labiatae/Lamiaceae] Typha latifolia L. [Typhaceae] Whitmania pigra Whitman [Hirudo]		
Yang 2012	QiMing granule	/	Astragalus aaronii	N	N

[Fabaceae/Leguminosae]

Pueraria montana var. Lobata

[Fabaceae/Leguminosae]

Rehmannia glutinosa

[Scrophulariaceae]

Lycium chinense

[Solanaceae Juss.]

Cassia obtusifolia L.

[Fabaceae/Leguminosae]

Leonurus japonicus Houtt.

[Labiatae/Lamiaceae]

Typha latifolia L.

[Typhaceae]

Whitmania pigra Whitman

			[Hirudo]		
Zheng 2014	QiMing granule	/	Astragalus aaronii<br>[Fabaceae/Leguminosae]<br>Pueraria montana var. Lobata<br>[Fabaceae/Leguminosae]<br>Rehmannia glutinosa<br>[Scrophulariaceae]<br>Lycium chinense<br>[Solanaceae Juss.]<br>Cassia obtusifolia L.<br>[Fabaceae/Leguminosae]<br>Leonurus japonicus Houtt.<br>[Labiatae/Lamiaceae]<br>Typha latifolia L.	N	N

			[Typhaceae]<br>Whitmania pigra Whitman<br>[Hirudo]		
Meng 2016	QiMing granule	/	Astragalus aaronii<br>[Fabaceae/Leguminosae]<br>Pueraria montana var. Lobata<br>[Fabaceae/Leguminosae]<br>Rehmannia glutinosa<br>[Scrophulariaceae]<br>Lycium chinense<br>[Solanaceae Juss.]<br>Cassia obtusifolia L.<br>[Fabaceae/Leguminosae]<br>Leonurus japonicus Houtt.	N	N

			[Labiatae/Lamiaceae]<br>Typha latifolia L.<br>[Typhaceae]<br>Whitmania pigra Whitman<br>[Hirudo]		
Wang 2018	QiMing granule	Zhejiang Wanma Pharmaceutical	Astragalus aaronii<br>[Fabaceae/Leguminosae]<br>Pueraria montana var. Lobata<br>[Fabaceae/Leguminosae]<br>Rehmannia glutinosa<br>[Scrophulariaceae]<br>Lycium chinense<br>[Solanaceae Juss.]<br>Cassia obtusifolia L.	N	N

			[Fabaceae/Leguminosae]<br>Leonurus japonicus Houtt.<br>[Labiatae/Lamiaceae]<br>Typha latifolia L.<br>[Typhaceae]<br>Whitmania pigra Whitman<br>[Hirudo]		
Feng 2016	QiMing granule	Zhejiang Wansheng Pharmaceutical Co.,Ltd	Astragalus aaronii<br>[Fabaceae/Leguminosae]<br>Pueraria montana var. Lobata<br>[Fabaceae/Leguminosae]<br>Rehmannia glutinosa<br>[Scrophulariaceae]<br>Lycium chinense	N	N

			[Solanaceae Juss.]<br>Cassia obtusifolia L.<br>[Fabaceae/Leguminosae]<br>Leonurus japonicus Houtt.<br>[Labiatae/Lamiaceae]<br>Typha latifolia L.<br>[Typhaceae]<br>Whitmania pigra Whitman<br>[Hirudo]		
Wang 2019	QiMing granule	Zhejiang Wanma Pharmaceutical Co., Ltd.	Astragalus aaronii<br>[Fabaceae/Leguminosae]<br>Pueraria montana var. Lobata<br>[Fabaceae/Leguminosae]<br>Rehmannia glutinosa	N	N

[Scrophulariaceae]

Lycium chinense

[Solanaceae Juss.]

Cassia obtusifolia L.

[Fabaceae/Leguminosae]

Leonurus japonicus Houtt.

[Labiatae/Lamiaceae]

Typha latifolia L.

[Typhaceae]

Whitmania pigra Whitman

[Hirudo]

Yan 2020	QiMing granule	Zhejiang Wansheng Pharmaceutical Co.,Ltd	Astragalus aaronii<br>[Fabaceae/Leguminosae]<br>Pueraria montana var. Lobata<br>[Fabaceae/Leguminosae]<br>Rehmannia glutinosa<br>[Scrophulariaceae]<br>Lycium chinense<br>[Solanaceae Juss.]<br>Cassia obtusifolia L.<br>[Fabaceae/Leguminosae]<br>Leonurus japonicus Houtt.<br>[Labiatae/Lamiaceae]<br>Typha latifolia L.<br>[Typhaceae]	N	N

Wang 2017	QiMing granule	/	Whitmania pigra Whitman<br>[Hirudo]<br>Astragalus aaronii<br>[Fabaceae/Leguminosae]<br>Pueraria montana var. Lobata<br>[Fabaceae/Leguminosae]<br>Rehmannia glutinosa<br>[Scrophulariaceae]<br>Lycium chinense<br>[Solanaceae Juss.]<br>Cassia obtusifolia L.<br>[Fabaceae/Leguminosae]<br>Leonurus japonicus Houtt.<br>[Labiatae/Lamiaceae]	N	N

Ge 2018	QiMing granule	Zhejiang Wansheng Pharmaceutical Co.,Ltd	Typha latifolia L.<br>[Typhaceae]<br>Whitmania pigra Whitman<br>[Hirudo]<br>Astragalus aaronii<br>[Fabaceae/Leguminosae]<br>Pueraria montana var. Lobata<br>[Fabaceae/Leguminosae]<br>Rehmannia glutinosa<br>[Scrophulariaceae]<br>Lycium chinense<br>[Solanaceae Juss.]<br>Cassia obtusifolia L.<br>[Fabaceae/Leguminosae]	N	N

			Leonurus japonicus Houtt.<br>[Labiatae/Lamiaceae]<br>Typha latifolia L.<br>[Typhaceae]<br>Whitmania pigra Whitman<br>[Hirudo]		
Yin 2018	QiMing granule	Zhejiang Wanma Pharmaceutical Co., Ltd.	Astragalus aaronii<br>[Fabaceae/Leguminosae]<br>Pueraria montana var. Lobata<br>[Fabaceae/Leguminosae]<br>Rehmannia glutinosa<br>[Scrophulariaceae]<br>Lycium chinense<br>[Solanaceae Juss.]	N	N

			Cassia obtusifolia L.<br>[Fabaceae/Leguminosae]<br>Leonurus japonicus Houtt.<br>[Labiatae/Lamiaceae]<br>Typha latifolia L.<br>[Typhaceae]<br>Whitmania pigra Whitman<br>[Hirudo]		
Wu 2015	QiMing granule	Zhejiang Wanma Pharmaceutical Co., Ltd.	Astragalus aaronii<br>[Fabaceae/Leguminosae]<br>Pueraria montana var. Lobata<br>[Fabaceae/Leguminosae]<br>Rehmannia glutinosa<br>[Scrophulariaceae]	N	N

			Lycium chinense<br>[Solanaceae Juss.]<br>Cassia obtusifolia L.<br>[Fabaceae/Leguminosae]<br>Leonurus japonicus Houtt.<br>[Labiatae/Lamiaceae]<br>Typha latifolia L.<br>[Typhaceae]<br>Whitmania pigra Whitman<br>[Hirudo]		
Mu 2018	QiMing granule	Zhejiang Wansheng Pharmaceutical Co.,Ltd	Astragalus aaronii<br>[Fabaceae/Leguminosae]<br>Pueraria montana var. Lobata<br>[Fabaceae/Leguminosae]	N	N

			Rehmannia glutinosa [Scrophulariaceae]<br>Lycium chinense [Solanaceae Juss.]<br>Cassia obtusifolia L. [Fabaceae/Leguminosae]<br>Leonurus japonicus Houtt. [Labiatae/Lamiaceae]<br>Typha latifolia L. [Typhaceae]<br>Whitmania pigra Whitman [Hirudo]		
Pang 2017	QiMing granule	/	Astragalus aaronii [Fabaceae/Leguminosae]	N	N

Pueraria montana var. Lobata

[Fabaceae/Leguminosae]

Rehmannia glutinosa

[Scrophulariaceae]

Lycium chinense

[Solanaceae Juss.]

Cassia obtusifolia L.

[Fabaceae/Leguminosae]

Leonurus japonicus Houtt.

[Labiatae/Lamiaceae]

Typha latifolia L.

[Typhaceae]

Whitmania pigra Whitman

[Hirudo]

Dai 2018	QiMing granule	/	Astragalus aaronii<br>[Fabaceae/Leguminosae]<br>Pueraria montana var. Lobata<br>[Fabaceae/Leguminosae]<br>Rehmannia glutinosa<br>[Scrophulariaceae]<br>Lycium chinense<br>[Solanaceae Juss.]<br>Cassia obtusifolia L.<br>[Fabaceae/Leguminosae]<br>Leonurus japonicus Houtt.<br>[Labiatae/Lamiaceae]<br>Typha latifolia L.<br>[Typhaceae]	N	N

Pan 2022	QiMing granule	Zhejiang Wansheng Pharmaceutical Co.,Ltd	Whitmania pigra Whitman<br>[Hirudo]<br>Astragalus aaronii<br>[Fabaceae/Leguminosae]<br>Pueraria montana var. Lobata<br>[Fabaceae/Leguminosae]<br>Rehmannia glutinosa<br>[Scrophulariaceae]<br>Lycium chinense<br>[Solanaceae Juss.]<br>Cassia obtusifolia L.<br>[Fabaceae/Leguminosae]<br>Leonurus japonicus Houtt.<br>[Labiatae/Lamiaceae]	N	N

Zhou 2015	QiMing granule	/	Typha latifolia L. [Typhaceae]<br>Whitmania pigra Whitman [Hirudo]<br>Astragalus aaronii [Fabaceae/Leguminosae]<br>Pueraria montana var. Lobata [Fabaceae/Leguminosae]<br>Rehmannia glutinosa [Scrophulariaceae]<br>Lycium chinense [Solanaceae Juss.]<br>Cassia obtusifolia L. [Fabaceae/Leguminosae]	N	N

Fan 2018	QiMing granule	/	Leonurus japonicus Houtt.<br>[Labiatae/Lamiaceae]<br>Typha latifolia L.<br>[Typhaceae]<br>Whitmania pigra Whitman<br>[Hirudo]<br>Astragalus aaronii<br>[Fabaceae/Leguminosae]<br>Pueraria montana var. Lobata<br>[Fabaceae/Leguminosae]<br>Rehmannia glutinosa<br>[Scrophulariaceae]<br>Lycium chinense<br>[Solanaceae Juss.]	N	N

			Cassia obtusifolia L.<br>[Fabaceae/Leguminosae]<br>Leonurus japonicus Houtt.<br>[Labiatae/Lamiaceae]<br>Typha latifolia L.<br>[Typhaceae]<br>Whitmania pigra Whitman<br>[Hirudo]		
Zhang 2016	QiMing granule	Zhejiang Wanma Pharmaceutical Co., Ltd.	Astragalus aaronii<br>[Fabaceae/Leguminosae]<br>Pueraria montana var. Lobata<br>[Fabaceae/Leguminosae]<br>Rehmannia glutinosa<br>[Scrophulariaceae]	N	N

	Lycium chinense<br>[Solanaceae Juss.]<br>Cassia obtusifolia L.<br>[Fabaceae/Leguminosae]<br>Leonurus japonicus Houtt.<br>[Labiatae/Lamiaceae]<br>Typha latifolia L.<br>[Typhaceae]<br>Whitmania pigra Whitman<br>[Hirudo]

*,The manufacturer of Qiming granule drugs is now named Zhejiang Sansheng Mandi Pharmaceutical Co., LTD., Zhejiang Wansheng Pharmaceutical Co., Ltd. and Zhejiang Wanma Pharmaceutical Co., Ltd. are the former names of Qiming granule manufacturers.

5. Sensitivity analysis

The results of the sensitivity analysis of the effect of QMKL vs CT in Overall efficacy

Study	P	I^2(%)	RR	95%CI
Fan 2018	<0.00001	0	1.42	[1.30,1.55]
Fang 2022	<0.00001	12	1.43	[1.31,1.56]
Kong 2015	<0.00001	19	1.48	[1.36,1.63]
Yang 2012	<0.00001	34	1.44	[1.32,1.57]
Zang 2011	<0.00001	15	1.49	[1.36,1.63]
Zheng 2016	<0.00001	36	1.46	[1.34,1.60]

The results of the sensitivity analysis of the effect of QMKL vs CT in Retinal circulation time

Study	P	I^2(%)	MD	95%CI
Kong 2015	0.09	/	-0.54	[-1.16,0.08]
Zheng 2016	0.07	/	-0.59	[-1.23,0.05]

The results of the sensitivity analysis of the effect of QMKL vs CT in Macular thickness

Study	P	I^2(%)	MD	95%CI
Fan 2018	0.04	0	-6.35	[-12.56,-0.15]
Kong 2015	0.13	89	-13.53	[-31.22,4.17]
Zheng 2016	0.02	80	-15.81	[-29.34,-2.27]

The results of the sensitivity analysis of the effect of QMKL+CT vs CT in Overall efficacy

Study	P	I^2(%)	RR	95%CI
Cao 2014	<0.00001	0	1.29	[1.25,1.33]
Chen 2016	<0.00001	0	1.28	[1.24,1.33]
Ge 2018	<0.00001	0	1.29	[1.24,1.33]
Huang 2017	<0.00001	0	1.29	[1.25,1.34]
Meng 2016	<0.00001	0	1.28	[1.24,1.33]
Sui 2014	<0.00001	0	1.28	[1.24,1.33]
Wang 2017	<0.00001	0	1.29	[1.24,1.33]
Wang 2018	<0.00001	0	1.28	[1.24,1.33]
Wang 2019	<0.00001	0	1.29	[1.25,1.34]
Yan 2020	<0.00001	0	1.28	[1.24,1.33]
Yang 2013	<0.00001	0	1.28	[1.24,1.33]
Yin 2018	<0.00001	0	1.28	[1.24,1.33]
Yin 2019	<0.00001	0	1.29	[1.24,1.33]
Yue 2016	<0.00001	0	1.27	[1.23,1.32]
Zhang 2013	<0.00001	0	1.28	[1.24,1.32]
Zhang 2015	<0.00001	0	1.29	[1.25,1.34]
Zheng 2014	<0.00001	0	1.29	[1.24,1.33]
Zhou 2017	<0.00001	0	1.29	[1.24,1.33]

Wu2015	<0.00001	0	1.29	[1.24,1.33]
Mu2018	<0.00001	0	1.29	[1.25,1.34]
Pang2017	<0.00001	0	1.28	[1.24,1.33]
Dai2018	<0.00001	0	1.28	[1.28,1.33]
Zhang2016	<0.00001	0	1.28	[1.24,1.33]
Pan2022	<0.00001	0	1.29	[1.24,1.33]
Zhou2015	<0.00001	0	1.28	[1.24,1.33]

The results of the sensitivity analysis of the effect of QMKL+CT vs CT in Visual acuity

Study	P	I^2(%)	MD	95%CI
Feng 2016	<0.00001	58	0.14	[0.09,0.19]
Sui 2014	<0.00001	54	0.14	[0.10,0.19]
Wang 2015	<0.00001	56	0.14	[0.10,0.19]
Yan 2020	<0.00001	45	0.15	[0.12,0.19]
Yin 2019	<0.00001	0	0.12	[0.09,0.15]
Zhou 2017	<0.00001	57	0.14	[0.10,0.17]

The results of the sensitivity analysis of the effect of QMKL+CT vs CT in Macular thickness

Study	P	I^2(%)	MD	95%CI
Cao 2014	<0.00001	83	-16.61	[-23.77,-9.45]
Ge 2018	<0.00001	81	-17.14	[-23.61,-10.67]
Yin 2019	0.006	82	-12.14	[-20.81,-3.48]
Zhou 2017	0.01	90	-12.59	[-22.72,-2.47]

The results of the sensitivity analysis of the effect of QMKL+CT vs CT in TG

Study	P	I^2(%)	MD	95%CI
Feng 2016	0.001	57	-0.24	[-0.38,-0.09]
Sui 2014	0.002	61	-0.22	[-0.36,-0.08]
Wang 2015	0.001	58	-0.23	[-0.38,-0.09]
Yan 2020	0.02	60	-0.18	[-0.32,-0.03]
Pang2017	0.02	0	-0.15	[-0.28,-0.02]

The results of the sensitivity analysis of the effect of QMKL+CT vs CT in TC

Study	P	I^2(%)	MD	95%CI
Feng 2016	0.007	90	-0.72	[-1.23,-0.20]
Sui 2014	0.08	97	-0.61	[-1.29,0.08]
Yan 2020	0.07	97	-0.60	[-1.24,0.04]

Pang2017	0.008	70	-0.32	[-0.56,-0.08]

The results of the sensitivity analysis of the effect of QMKL+CT vs CT in HDL-C

Study	P	I^2(%)	MD	95%CI
Feng 2016	0.88	91	-0.03	[-0.41,0.35]
Sui 2014	0.51	73	0.08	[-0.16,0.31]
Wang 2015	0.96	90	-0.01	[-0.40,0.38]
Yan 2020	0.33	82	-0.14	[-0.42,0.14]
Pang2017	0.58	89	-0.09	[-0.43,0.24]

The results of the sensitivity analysis of the effect of QMKL+CT vs CT in LDL-C

Study	P	I^2(%)	MD	95%CI
Feng 2016	0.01	70	-0.46	[-0.81,-0.11]
Sui 2014	0.03	75	-0.43	[-0.82,-0.05]
Wang 2015	0.07	79	-0.39	[-0.81,0.03]
Yan 2020	0.11	55	-0.23	[-0.52,0.05]
Pang2017	0.17	76	-0.30	[-0.74,0.13]

The results of the sensitivity analysis of the effect of QMKL+CT vs CT in HbA1c

Study	P	I^2(%)	MD	95%CI
Feng 2016	0.20	0	-0.27	[-0.68,0.14]
Sui 2014	0.55	0	-0.15	[-0.63,0.33]
Yan 2020	0.27	0	-0.26	[-0.73,0.20]

6. Abbreviations

QM	QiMing granules
DR	diabetic retinopathy
RCTs	randomized controlled trials
TCM	traditional Chinese medicine
RoB 2.0	Cochrane risk-of-bias tool for randomized trials 2.0
TG	triglyceride
TC	total cholesterol
LDL-C	low-density lipoprotein cholesterol
HDL-C	high-density lipoprotein cholesterol
HbA1c	glycated hemoglobin
MD	mean difference
RR	risk ratio
CI	confidence interval
CT	conventional treatment
S	second
μm	micrometre
D	diopter

mmol	millimoles concentration
PRISMA	Preferred Reporting Items for Systematic Reviews and Meta-Analyses
PICO(S)	participants, intervention, control, outcomes (study designs)
GRADE	the Grading of Recommendation, Assessment, Development, and Evaluation
T	treatment group
C	control group
m	months
NO.S	numbers of studies
VEGF	vascular endothelial growth factor
TGF- β	transforminggrowthfactor- β
TNF	tumor necrosis factor
VEGFA	vascular endothelial growth factor A
NF-kB	nuclear factor kappa-B
